# Supplementary material for: Incidence and transmission of respiratory syncytial virus in urban and rural South Africa, 2017-2018
Source: Nat Commun. 2024 Jan 2;15:116. doi: 10.1038/s41467-023-44275-y (PMC10761814; doi:10.1038/s41467-023-44275-y)
Supplement: Supplementary file 1 — Supplementary Information [file 41467_2023_44275_MOESM1_ESM.pdf]

Supplementary material

Article title: Incidence and transmission of respiratory syncytial virus in urban and rural South Africa, 2017-2018

Supplementary methods

Household selection

We selected households using different methods in each site. In Agincourt (the rural site), each year we selected two villages out of 29 within the HDSS according to convenience considering proximity and burden of other studies within the site. Within these villages, households known to have >2 members from a census conducted in the previous year or more recently, were randomly selected. Simple random sampling was performed using a complete list of all households with >2 members within the selected villages. In Jouberton Township (the urban site), a list of 450 random global positioning system (GPS) coordinates were generated in the study area using Google Earth as previously described.[34] Study staff navigated to the coordinates and selected the nearest house within 30 meters of the location. If there was no dwelling within 30m the coordinates were discarded.

Participant enrolment

Participants were enrolled during November and December of the year preceding the period of active follow up i.e. during 2016 for the 2017 cohort and during 2017 for the 2018 cohort. Study staff confirmed that the household contained >2 members and requested permission from the head of household to inform members about the study. If the head of household was unavailable after a minimum of 3 attempts or was a minor then the house was excluded. Informed consent to participate in the study was requested from all household members aged ≥18 years, assent from children aged 7 to 17 years, and consent from a parent or guardian for children younger than 18 years. Individuals with <11 completed follow up visits were also excluded from analysis. Within included households, when compared to individuals who did not participate in the study, individuals who participated were more likely to be female and less likely to be aged 15-44 years.[35] A comparison of characteristics of included households to the communities from which they were sampled has been previously published.[35]

Data collection

To ensure that we captured the start and end of each year's RSV season at each site, twice-weekly follow up was from January through October. The RSV season usually occurs in autumn from

February through May and precedes the annual winter influenza season. Intensive follow-up was stopped during November through December to allow for enrolment of the next years cohort. Households who withdrew from the study during January-April were replaced by a new household for the remaining follow-up period.

Symptom data for children was consistently collected from the individual identified as primary caregiver at the time of enrolment. Challenges with quality of symptom data collected in 2016 (data from 2016 not included in this study) have been previously described.[9] This resulted in implementation of a number of measures to improve quality of symptom data including simplifying the symptom form to require the same data fields irrespective of symptom reporting, refresher training for field workers on symptom data collection every 1-2 months, reminding participants at least monthly of the importance of consistently and validly reporting symptoms despite fatigue, encouraging field staff to observe participants for the presence of visible clinical signs such as cough or runny nose and probe participants if not reported and conducting regular supervisory visits with focus on quality of symptom data collection.

Refresher training was provided to field workers on different aspects of study implementation including data collection, specimen collection and use of online databases at least monthly. Site supervisors conducted regular (at least monthly) supervisory visits to assess study implementation in the field and external (teams from outside the study sites) supervisory visits were conducted quarterly.

Definitions and measurements

Current tuberculosis was defined as an individual currently receiving treatment for tuberculosis. Previous tuberculosis was defined as a self-reported history of having been diagnosed with or treated for tuberculosis. All individuals reporting cough, weight loss or night sweats for >2 weeks were offered tuberculosis testing and any individuals testing positive during the study were classified as having current tuberculosis.

Participants were considered people living with HIV (PLWH) if they had a documented positive HIV result or evidence of antiretroviral treatment use and HIV-uninfected if they had a documented negative HIV result in the 6 months prior to enrolment. Individuals with unknown HIV status were offered HIV testing and those newly diagnosed with HIV were referred for assessment and initiation of antiretroviral treatment.

Infants were defined as HIV exposed but uninfected if they were HIV-uninfected but the mother was HIV-infected. There were only two included infants known to be born to HIV-infected mothers, therefore associations with HIV exposure were not further investigated. For HIV-infected individuals, specimens were collected for CD4+ T cell and HIV quantitative viral load testing at diagnosis or enrolment.

Sex was determined based on self-report. Sex was considered as a potential exposure of interest in the study. For each outcome of interest results are reportedly separately by sex.

Written vaccination history was obtained for all children aged <5 years from patient-held immunisation records and, if needed, vaccination records at health facilities. Primary caregivers giving a history of the child never being vaccinated were recorded as unvaccinated.

Household income was evaluated through self-reporting by the head of household. For households where income varied from month to month, the head of household was asked to provide the average monthly household income over the previous 12 months.

Indoor respirable particulate matter (PM_4) mass concentrations were measured gravimetrically using filter-based sampling. Mixed cellulose ester filters were exposed daily for 24 hours in each household, for a period of one week, during both summer and winter. Indoor temperature was continuously monitored using Thermochorn iButton DS1922L sensors.

Quantitative urine cotinine tests were performed using the IMMULITE® 1000 Nicotine Metabolite Assay Kit (Siemens Medical Solutions Diagnostics, Gly Rhonwy, UK). HIV testing was offered to all participants on enrolment.

Laboratory methods

The FTD Flu/RSV detection assay (Fast Track Diagnostics, Luxembourg) has a reported sensitivity of 100% and a specificity of 100% according to a clinical validation study of 60 samples reported by the manufacturer.[36] All positive samples were sent for subgrouping and the subgroup was determined for 79% (723/917) of positive samples, undetermined subgroup was likely because the viral load was too low. Determination of subgroup was considered a second confirmatory test for the presence of RSV-specific RNA in the sample. All samples for which subgroup could not be determined, were

retested for RSV by real-time reverse transcription polymerase chain reaction (rRT-PCR) using the FTD Flu/RSV detection assay (Fast Track Diagnostics, Luxembourg) twice from a separate extract. Samples which were positive for ≥2 of 3 tests were included as positive.

Definitions and statistical analyses

For rRT-PCR positivity where a subgroup was not determined we assigned a subgroup if the individual had a confirmed subgroup of the same RSV type (A or B) within the two preceding or following visits (Supplementary figure 2). If a subgroup could not be assigned using the described criterion, we considered an episode of infection RSV untyped. For the analyses of shedding duration, HCIR, generation interval, and comparison of index cases to non-index cases, we included mixed subgroup infections as two separate infections. For the analysis of incidence and symptomatic fraction, we counted mixed infections only once.

The generation interval was calculated as the difference between the dates of the first positive PCR tests in the index case and in the secondary case, each adjusted by adding a random number selected from a uniform distribution between 0 and 3 (inclusive). For the analysis of generation interval and HCIR, we included all secondary cases with PCR positivity <17 days after the index case. This was chosen as the 75% quantile of the of the generation intervals observed in our cohort was 16 days (Figure 3). Infection episodes after this period are more likely to be tertiary or quaternary transmission events. As index case characteristics associated with transmission parameters was a focus of our analysis, we tried to limit the analysis to early transmissions in the household.

For the analysis of factors associated with incidence we assessed overdispersion starting with a negative binomial model and retained a simpler Poisson model as there was no statistically significant overdispersion detected. For the analyses by age group the age group with the lowest point estimates and sufficient numbers were chosen as the reference to present odds ratios greater than 1 for the other relevant groups. For models including age group of both the index case and household contact, we used the same reference group for both. Multivariable models were built using forward and backward selection. Age was included in all models a priori. For the model of factors associated with symptoms, age, HIV status and duration of shedding were associated with the outcome on univariate analysis at p<0.2. HIV fell out of the final model because it was confounded by age. For the model of factors associated with duration of shedding, minimum Ct value and number of symptoms were associated with the outcome on univariate analysis and retained in the final model. On univariate analysis subgroup untyped was associated with duration of

shedding at p<0.2 but this fell out of the final model as it was confounded by cycle threshold value (ie untyped samples had high cycle threshold). For the model of HCIR, number of symptoms and duration of shedding were associated with the outcome on univariate analysis and retained in the final model. Minimum Ct value fell out of the model on multivariable analysis, because of confounding by shedding duration and number of symptoms. Similarly, HIV fell out of the model because of confounding by age. For the model of factors associated with RSV incidence, year and age were associated with the outcome on univariate analysis and retained in the final model. HIV status fell out of the final model because it was confounded by age. For the model of factors associated with generation interval age and subgroup remained in the final model. Underlying illness and duration of shedding were associated on univariate analysis but fell out of the final model as they were confounded by age.

We used Weibull accelerated time failure regression to estimate the factors associated with shedding duration and generation interval. Weibull regression implements a fully parametric model (as compared with semi-parametric model implemented by the Cox proportional hazard model). Advantages of parametric models in survival analysis include: (i) full maximum likelihood can be used to estimate parameters, and (ii) estimated parameters provide clinically meaningful estimates of effect. In Weibull regression the distribution of time to event, T, as a function of single covariate is written as:

$$In(T)=\beta_0+\beta_1X+\sigma\varepsilon$$

where β1 is the coefficient for corresponding covariate, ε follows extreme minimum value distribution G(0, σ) and σ is the shape parameter.

Sensitivity analyses

In order to explore possible bias introduced by the fact that in some households, not all members participated in the study, we performed a sensitivity analysis restricted to households with all members participating. To explore the effect of our definition of time between new episodes we performed sensitivity analyses reducing the time between episodes to >1 week and increasing it to >3 weeks. To explore the effect of our cut-off of 17 days for analysis of generation interval and HICR, we performed sensitivity analyses including all subsequent cases in the household irrespective of interval.

Ethics

The U.S. Centers for Disease Control and Prevention's Institutional Review Board relied on the local review in accordance with applicable federal law and CDC policy (25 C.F.R. part 46; 21 C.F.R. part 56).

Supplementary results

Clusters per household and numbers per cluster

There was an average of 1.4 clusters and 1.7 infected individuals per infected household (Supplementary table 2).

Sensitivity analyses

Of 225 included households, 151 households (with 717 household members) had all household members included in the study. On sensitivity analysis restricted to this subset of households, HCIR was similar to the main analysis (11.5%, 62/541). Factors associated with HCIR were similar to the main analysis in magnitude and direction, but the association with age of the household member was no longer statistically significant, likely as a result of low numbers (data not shown in supplement, results of this analysis, together with analysis code available at https://github.com/crdm-nicd/phirst_rsv). The proportion of infection episodes acquired in the community was similar on sensitivity analysis (68%, 154/228).

Reducing the interval between episodes to 1 week did not have any effect on the number of episodes or episode duration i.e. number of episodes remained at 400 with duration of 6.7 days as in the original analysis. Increasing the time between episodes to 3 weeks dropped the number of episodes to 393 and increased the mean episode duration to 7.1 days (standard deviation 6.4, range 3-53 days). On this analysis the median generation interval changed marginally to 8.4 days (standard deviation 3.6, range 3-16). Given the small change in episode numbers on these analyses, there was no notable difference in direction and magnitude of factors associated with HCIR, shedding duration or serial interval (data not shown in supplement, results of this analysis, together with analysis code available at https://github.com/crdm-nicd/phirst_rsv).

On sensitivity analysis not applying any restriction on generation interval, the mean generation interval was 11.0 days (standard deviation 6.9 days, range 1 to 35 days) and the overall HCIR was 14% (135 of 954 exposed household members). On multivariable analysis controlling for index case age, factors associated with increased transmission were similar to the main analysis in magnitude and direction (data not shown in supplement, results of this analysis, together with analysis code available at https://github.com/crdm-nicd/phirst_rsv).

1

Supplementary Table 1: Table of outcomes and definitions

Outcome	Definition	Restrictions
RSV infection episode	One or more nasopharyngeal swab rRT-PCR positive (cycle threshold (Ct) value <37) for RSV. We considered a new infection when the individual tested positive for a different subgroup or the same subgroup >2 weeks from the last day of the last previous positive; else, we considered it the same episode.	Mixed infections counted only once.
Episode/shedding duration (days)	The first to the last day of rRT-PCR positivity plus a random number from a uniform distribution between 0 and 3 (inclusive) for the start and end of the episode to account for the midpoint time from subsequent visits. Episode duration for single visit positives was estimated by selecting a random number from a uniform distribution between 0 and 6 (inclusive).	Included mixed subgroup infections as two separate infections.
RSV illness episode	An infection episode with ≥1 symptom reported from one visit before to one visit after the RSV infection episode.	Mixed infections counted only once.
Symptomatic fraction	Number of illness episodes divided by number of infection episodes.	Mixed infections counted only once.
Incidence	Number of episodes divided by the person time under observation reported per 100 person years.	Mixed infections counted only once.
Cluster	All infections of the same subgroup within a household within an interval between infections of ≤2 mean serial intervals (3.5 days), including single infections.	Included mixed subgroup infections as two separate infections.
Cluster duration	The interval from the first day of positivity of the first individual in a cluster to the last day of positivity of the last individual. First and last day adjusted by adding a random number from a uniform distribution between 0 and 3 (inclusive) for the start and end of the episode (same value from episode duration calculation) to account for the mid-point time from subsequent visits.	

Index case	First individual testing positive within a cluster.	
Coprimary index cases	When more than one individual in a cluster tested positive on the first visit.	
Household cumulative infection risk (HCIR)	The cumulative number of all household members with RSV infection detected within a household cluster, divided by the total number of individuals participating in the study in the affected household, exclusive of the index case.	Restricted to clusters without coprimary index cases. Restricted to secondary cases with first RSV positive <17 days after the index case first positive. Included mixed subgroup infections as two separate infections.
Percent of infections acquired in the community	Total number of index case infections divided by total number of infections.	Restricted to clusters without coprimary index cases.
Generation interval	Difference between the dates of the first positive PCR tests in the index case and in the secondary case each adjusted by adding a random number selected from a uniform distribution between 0 and 3 (inclusive).	Restricted to secondary cases who were PCR positive <17 days after the index case. Included mixed subgroup infections as two separate infections.

Supplementary Table 2: Baseline characteristics of households and individuals included in PHIRST at a rural and an urban site, South Africa, 2017-2018

Characteristic	Overall n (%) or median (IQR)	Rural n (%) or median (IQR)	Urban n (%) or median (IQR)	p[k]
**Household level characteristics**	**N=225**	**N=109**	**N=116**	
Intensive follow-up year				
2017	108 (48)	53 (49)	55 (47)	0.86
2018	117 (52)	56 (51)	61 (53)	
Number of household members				
3-5	143 (64)	67 (61)	76 (66)	Ref
6-10	72 (33)	38 (35)	37 (32)	0.59
>10	7 (3)	4 (4)	3 (3)	0.60
Number of household members	5 (3-10)	5 (3-10)	5 (3-10)	0.44
Number of rooms				
1-4	99 (44)	47 (43)	52 (45)	Ref
5-9	117 (52)	57 (52)	60 (52)	0.86
≥10	9 (4)	5 (5)	4 (3)	0.64
Number of rooms	5 (2-9)	5 (1-9)	5 (2-9)	0.69
Number of rooms for sleeping				
1-2	127 (56)	58 (53)	69 (59)	Ref
3-4	93 (41)	48 (44)	45 (39)	0.38
>4	5 (2)	3 (3)	2 (2)	0.53
Number of rooms for sleeping	2 (1-4)	2 (1-4)	2 (1-4)	0.42
Crowding (people/sleeping room)				
≤2	115 (51)	52 (48)	63 (54)	0.32
>2	110 (49)	57 (52)	53 (46)	
Child aged <5 years in house				
Yes	153 (68)	96 (88)	57 (49)	<0.001
No	72 (32)	12 (12)	59 (51)	
Household member smokes indoors				
Yes	44 (20)	9 (8)	35 (30)	<0.001
No	181 (80)	100 (92)	81 (70)	
Main water source				
Tap outside	115 (51)	57 (52)	58 (50)	0.73
Tap inside	110 (49)	52 (48)	58 (50)	
Handwashing place with water in house				

Yes	182 (81)	69 (63)	113 (97)	<0.001
No	43 (19)	40 (37)	3 (3)	
Main fuel for cooking				
Electricity	183 (82)	74 (68)	109 (95)	<0.001
Wood	36 (16)	35 (32)	1 (1)	Ref
Paraffin/gas/other	5 (2)	0 (0)	5 (4)	NE
Monthly household income[a]				
≤R800 (≤USD54)	28 (13)	15 (14)	13 (11)	Ref
R801-R1600 (USD55-108)	64 (29)	30 (29)	34 (30)	0.56
R1601-R3200 (USD109-116)	71 (32)	38 (36)	33 (29)	1.00
R3201-R6400 (USD117-232)	44 (20)	17 (16)	27 (24)	0.22
R6401-R12800 (USD233-464)	8 (4)	5 (5)	3 (3)	0.66
>R12800 (>USD464)	4 (2)	0 (0)	4 (4)	NE
Summer indoor PM4				
>25 µg/m^{-3}[b]	89 (46)	57 (61)	32 (32)	<0.001
≤25 µg/m^{-3}[b]	104 (54)	37 (39)	67 (68)	
Winter indoor PM4>25µg/m^{-3}[b]				
>25 µg/m^{-3}[b]	152 (78)	60 (63)	92 (92)	<0.001
≤25 µg/m^{-3}[b]	44 (22)	36 (38)	8 (8)	
Indoor summer temperature °C[c]	22 (19-25)	24 (21-25)	21 (19-23)	<0.001
Indoor winter temperature °C[c]	16 (9-20)	18 (16-20)	12 (8-16)	<0.001
**Individual level characteristics**	**N=1,116**	**N=561**	**N=555**	
Age group (years)				
<1	22 (2)	9 (2)	13 (2)	0.03
1-4	158 (14)	104 (19)	54 (10)	Ref
5-12	302 (27)	166 (30)	136 (25)	0.05
13-18	161 (14)	84 (15)	77 (14)	0.01
19-44	291 (26)	124 (22)	167 (30)	<0.001
45-64	137 (12)	52 (9)	85 (15)	<0.001
≥65	45 (4)	22 (4)	23 (4)	0.04
Female sex				
Female	680 (61)	358 (64)	322 (58)	<0.001
Male	436 (39)	203 (36)	233 (42)	
Year 2018				
2017	558 (50)	285 (51)	273 (49)	0.30
2018	558 (50)	276 (49)	282 (51)	
Level of education[d]				

No schooling	52 (11)	42 (21)	10 (4)	Ref
Primary schooling	111 (23)	50 (25)	61 (22)	0.001
Some secondary	183 (38)	44 (22)	139 (49)	0.30
Secondary completed	123 (25)	62 (31)	61 (22)	0.52
Post-secondary	16 (3)	5 (2)	11 (4)	0.16
Employment[d]				
Unemployed	272 (56)	131 (65)	141 (50)	Ref
Employed	183 (38)	56 (28)	127 (45)	<0.001
Student	30 (6)	16 (8)	15 (5)	0.59
Reported alcohol use[e]				
Yes	217 (37)	37 (15)	180 (54)	<0.001
No	362 (63)	211 (85)	151 (46)	
Reported current cigarette smoking[e]				
Yes	91 (16)	11 (4)	80 (24)	<0.001
No	488 (84)	237 (96)	251 (76)	
Reported current snuff smoking[e]				
Yes	63 (11)	3 (1)	60 (18)	<0.001
No	516 (89)	245 (99)	271 (82)	
Reported current any smoking[e]				
Yes	157 (27)	14 (6)	143 (43)	<0.001
No	422 (73)	234 (94)	188 (57)	
Smoke inside[f]				
Yes	56 (36)	2 (14)	54 (38)	0.10
No	101 (64)	12 (86)	89 (62)	
Urine cotinine (all ages)[g]				
Negative	437 (41)	356 (65)	81 (15)	Ref
Passive exposure	466 (44)	169 (31)	297 (56)	<0.001
Active smoking	167 (16)	19 (3)	148 (28)	<0.001
Unknown	46	17	29	
HIV status[h]				
Uninfected	908 (84)	485 (88)	423 (81)	Ref
Infected	167 (16)	68 (12)	99 (19)	0.003
Unknown	41	8	33	
ART use among HIV-infected				
Currently receiving	142 (85%)	55 (81)	87 (88)	0.44
Not receiving	18 (11%)	9 (13)	9 (9)	
Not reported	7 (4)	4 (6)	3 (3)	

HIV viral suppression among individuals receiving ART				
Suppressed throughout	53 (37)	16 (29)	37 (43)	0.01
Became suppressed during study	27 (19)	18 (33)	9 (10)	
Suppressed at some point	6 (4)	3 (5)	3 (3)	
Never suppressed	40 (28)	15 (27)	25 (29)	
No viral load results	16 (11)	3 (5)	13 (15)	
Previous tuberculosis	57 (5)	11 (2)	46 (8)	<0.001
Current tuberculosis	18 (2)	1 (<1)	17 (3)	0.01
Other underlying illness[i]	27 (2)	1 (<1)	26 (5)	0.001
Influenza vaccination	1 (<1)	0 (0)	1 (<1)	NE
Pneumococcal vaccine up to date for age[j]				
Yes	150 (96)	95 (98)	55 (93)	Ref
No	6 (4)	2 (2)	4 (7)	0.16
No data	24	16	8	
DTaP-IPV/Hib vaccine up to date for age[i]				
Yes	152 (97)	95 (98)	57 (95)	Ref
No	5 (3)	2 (2)	3 (5)	0.32
No data	23	16	7	

DTaP-IPV/Hib – Diphtheria, tetanus, acellular pertussis, inactivated polio, *Haemophilus influenzae* type B vaccine, IQR – interquartile range, OR – odds ratio, CI – confidence interval, NE – not estimated, n - number, USD – United States Dollar, ART - antiretroviral treatment

[a]Data available for 219 households, 105 rural and 114 urban [b]Median respirable particulate matter over 7 day sampling period available for 193 households 94 rural and 99 urban. [c]Median indoor temperature over 7 day sampling period in degrees centigrade available for 196 households 96 rural and 100 urban. [d]Individuals aged ≥18 years N=485, 203 at rural site and 282 at urban site [e]Individuals aged ≥15 years N=579, 248 at rural site and 331 at urban site [f]Among those reporting any current smoking [g]% and p value among individuals with known urine cotinine status, all individuals were eligible for urine cotinine testing [h]Among 141 HIV-infected individuals with available CD4+ T cell count data, 102 (72%) had CD4+ T cell counts >500/µl (36 at rural site, 66 at urban site), 31 (22%) were 200-500/µl (22 at rural site, 9 at urban site) and 8 (6%) were <200/µl (4 at each site). Two infants known to be born to HIV-infected mothers (HIV exposed) [i]Self-reported history of asthma, lung disease, heart disease, stroke, spinal cord injury, epilepsy, organ transplant, immunosuppressive therapy, organ transplantation, cancer, liver disease, renal disease or diabetes [j]Individuals aged <5 years N=180, 113 at rural site and 67 at urban site, 229 with available vaccination data, 139 at the rural site and 90 at the urban site

[k]p value comparing characteristics of households and individuals between the urban and rural site using logistic regression adjusted for clustering by site and household

Supplementary table 3: Proportion of households testing RSV positive, number of clusters and mean cluster size, attack rate and rates of RSV infections per 100 person-years by year and site, at a rural and an urban site, South Africa, 2017-2018

Year	Site	Household level			Individual level				
		Proportion of households with at least one person testing RSV positive n/N (%)	Clusters per infected household Mean (Range) [a]	Infected individuals per household with at least one infected individual Mean (Range)[b]	Person years of follow up	At least one infection episode [c]	Rate per 100 person-years (at least one episode) (95% CI) [c]	Total infection episodes [d]	Rate per 100 person years (multiple episodes) (95% CI) [d]
2017-2018	Rural and urban	168/225 (75)	1.4 (1-4)	1.7 (1-9)	839.1	359	42.8 (38.6-47.5)	396	47.2 (42.8-52.1)
2017-2018	Rural	83/109 (76)	1.5 (1-4)	1.7 (1-9)	421.4	186	44.1 (38.2-60.0)	207	49.1 (42.9-56.3)
2017-2018	Urban	85/116 (73)	1.4 (1-4)	1.7 (1-5)	417.6	173	41.4 (35.7-48.1)	189	45.3 (39.2-52.2)
2017	Rural and urban	78/108 (72)	1.4 (1-4)	1.8 (1-5)	421.4	167	39.6 (34.0-46.1)	181	43.0 (37.1-49.7)
2017	Rural	39/53 (74)	1.4 (1-3)	1.7 (1-5)	214.9	81	37.7 (30.3-46.8)	86	40.0 (32.4-49.4)
2017	Urban	39/55 (71)	1.4 (1-4)	1.9 (1-5)	206.3	86	41.7 (33.7-51.5)	95	46.0 (37.7-56.3)
2018	Rural and urban	90/117 (77)	1.5 (1-4)	1.7 (1-9)	417.8	192	46.0 (39.9-52.9)	215	51.5 (45.0-58.8)
2018	Rural	44/56 (79)	1.7 (1-4)	1.8 (1-9)	206.4	105	50.9 (42.0-61.6)	121	58.6 (49.1-70.0)
2018	Urban	46/61 (75)	1.3 (1-3)	1.6 (1-5)	211.3	87	41.1 (33.4-50.8)	94	44.5 (36.3-54.5)

[a]Among 168 households experiencing at least 1 cluster of RSV infection, 109 (65%) had 1 cluster, 47 (28%) had 2 clusters, 9 (5%) had 3 clusters and 3 (2%) had 4 clusters [b]Among 242 clusters of RSV infection, 147 (61%) involved only 1 infected individual within the household [c]Individuals testing RSV positive at least once during follow up counted once, incidence estimated as number of episodes divided by the person time under observation [d]Includes repeat episodes, among 359 individuals experiencing at

least one RSV infection episode, 31 (9%) had a second RSV infection and 3 (1%) had 3 RSV infections within the same year, incidence estimated as number of episodes divided by the person time under observation. Among the 396 episodes, includes 4 episodes of mixed infection (RSV A and B).

CI – confidence interval, N-number

Supplementary table 4: Rates of RSV infections and RSV-associated illness per 100 person-years by age group, at a rural and an urban site, South Africa, 2017-2018

Age group (years)	**Infections** Person years of follow up	At least one episode [a]	Rate (at least one episode) (95% CI) [a]	Total episodes [b]	Rate (multiple episodes) (95% CI) [b]	Repeat infections n/N (%)
<1	16.5	11	66.5 (36.8-120.1)	12	72.6 (41.2-127.8)	1/12 (8)
1-4	117.5	82	69.8 (53.2-868.6)	93	79.1 (64.6-97.0)	11/93 (12)
5-12	227.8	101	44.3 (36.5-53.9)	111	48.7 (40.4-58.7)	10/111 (9)
13-18	121.6	57	46.9 (36.2-60.8)	64	52.6 (41.2-67.2)	7/64 (11)
19-44	217.5	62	28.5 (22.2-36.6)	66	30.3 (23.8-38.6)	4/66 (6)
45-64	104.4	42	40.2 (29.7-54.4)	44	42.1 (31.3-56.6)	2/44 (5)
≥65	33.7	4	11.9 (4.4-31.6)	6	17.8 (13.4-56.6)	2/6 (33)
All ages	839	359	42.8 (38.6-47.5)	396	47.2 (42.8-52.1)	37/396 (9)

	**Any illness**					
	**≥1 symptom (%)**		**≥2 symptoms (any)(%)**		**ILI (Fever and cough)(%)**	
	**N[c](%)[d]**	**Rate (95% CI)**	**N[c](%)[d]**	**Rate (95% CI)**	**N[c](%)[d]**	**Rate (95% CI)**
<1	8 (66)	48.4 (24.1-96.7)	7 (58)	42.3 (20.2-88.8)	1 (8)	6.1 (0.9-42.9)
1-4	45 (48)	38.3 (28.6-51.3)	22 (24)	18.7 (12.3-28.4)	6 (6)	5.1 (2.3-11.4)
5-12	34 (31)	14.9 (10.7-20.9)	21 (19)	9.2 (6.0-14.1)	6 (5)	2.6 (1.2-5.9)
13-18	12 (19)	9.9 (5.6-17.4)	8 (13)	6.6 (3.3-13.2)	2 (3)	1.6 (0.4-6.6)
19-44	18 (27)	8.3 (5.2-13.1)	10 (15)	4.6 (2.5-8.5)	2 (3)	0.9 (0.2-3.7)
45-64	11 (25)	10.5 (5.8-19.0)	6 (14)	5.8 (2.6-12.8)	2 (5)	1.9 (0.5-7.7)
≥65	4 (67)	11.9 (4.5-31.6)	3 (50)	8.9 (2.9-27.6)	1 (17)	3.0 (0.4-21.1)
All ages	132 (33)	15.7 (13.3-18.7)	77 (19)	9.2 (7.3-11.5)	20 (5)	2.4 (1.5-3.7)

	**Medically attended illness**					
	**≥1 symptom**		**≥2 symptom**		**ILI (Fever and cough)(%)**	
	**N[c](%)[d]**	**Rate (95% CI)**	**N[c](%)[d]**	**Rate (95% CI)**	**N[c](%)[d]**	**Rate (95% CI)**
<1	1 (8)	6.1 (0.9-42.9)	1 (8)	6.1 (0.9-42.9)	1 (8)	6.1 (0.9-42.9)
1-4	4 (4)	3.4 (1.3-9.1)	3 (3)	2.6 (0.8-7.9)	2 (2)	1.7 (0.4-6.8)
5-12	2 (2)	0.9 (0.2-3.5)	2 (2)	0.9 (0.2-3.5)	0 (0)	0
13-18	0 (0)	0	0 (0)	0	0 (0)	0
19-44	0 (0)	0	0 (0)	0	0 (0)	0
45-64	2 (5)	1.9 (0.5-7.7)	2 (5)	1.9 (0.5-7.7)	0 (0)	0
≥65	0 (0)	0	0 (0)	0	0 (0)	0
All ages	9 (2)	1.1 (0.6-2.1)	8 (2)	1.0 (0.5-1.9)	3 (1)	0.4 (0.1-1.1)

ILI – Influenza-like illness. Incidence rate estimated as number of episodes divided by the person time under observation [a]Individuals testing RSV positive at least once during follow up counted once [b]Includes repeat episodes (Among 359 individuals who experienced at least one RSV episode in a season, 325 (91%) had 1 episode, 31 (9%) had 2 episodes and 3 (1%) had 3 episodes) [c]Number of episodes and percent of RSV infection episodes with ≥1 symptom, ≥2 symptoms or ILI (includes repeat episodes) [d]Percent of all episodes

Supplementary table 5: Factors associated with RSV incidence at a rural and an urban site, South Africa, 2017-2018*

		Including repeat RSV episode			Including only first episode		
**Variable**		**Rate per 100 person years [a] (95% CI)**	**Univariate RR**	**Multivariable RR**	**Rate per 100 person years [b] (95% CI)**	**Univariate RR**	**Multivariable RR**
Year	2017	43.0 (37.1-49.7)	Reference	Reference	39.6 (34.1-46.1)	Reference	Reference
	2018	51.5 (45.0-58.8)	1.2 (0.9-1.5)	1.2 (0.9-1.5)	46.0 (40.0-52.9)	1.2 (0.9-1.4)	1.2 (0.9-1.5)
Site	Rural	49.1 (42.9-56.3)	Reference		44.1 (38.2-51.0)	Reference	
	Urban	45.2 (39.2-52.1)	0.9 (0.7-1.2)		41.4 (36.0-48.1)	0.9 (0.8-1.2)	
Age group (years)	<1	72.6 (41.2-127-8)	2.4 (1.5-3.9)	2.4 (1.5-3.8)	66.5 (36.8-120.1)	2.3 (1.5-3.7)	2.4 (1.5-3.8)
	1-4	79.1 (64.6-97.0)	2.6 (2.0-3.4)	2.6 (2.0-3.5)	69.8 (56.2-86.6)	2.4 (1.9-3.2)	2.6 (2.0-3.5)
	5-12	48.7 (40.5-58.7)	1.6 (1.2-2.1)	1.6 (1.2-2.1)	44.3 (36.5-53.9)	1.6 (1.2-2.1)	1.6 (1.2-2.1)
	13-18	52.6 (41.2-67.2)	1.7 (1.3-2.3)	1.8 (1.3-2.4)	46.9 (36.2-60.8)	1.6 (1.2-2.2)	1.8 (1.3-2.4)
	19-44	30.3 (23.8-38.6)	Reference	Reference	28.5 (22.2-36.6)	Reference	Reference
	45-64	42.1 (31.4-56.6)	1.4 (0.9-2.0)	1.4 (1.1-2.0)	40.2 (29.7-54.4)	1.4 (1.1-2.0)	1.4 (1.1-2.0)
	≥65	17.8 (13.4-56.6)	0.6 (0.2-1.8)	0.6 (0.2-1.9)	11.9 (4.5-31.6)	0.4 (0.2-1.1)	0.6 (0.2-1.9)
Sex	Female	46.0 (40.5-52.3)	0.9 (0.8-1.1)		42.9 (36.3-50.6)	1.0 (0.8-1.2)	
	Male	48.9 (41.9-57.2)	Reference		42.7 (37.4-48.8)	Reference	
HIV	Infected	38.4 (28.9-51.0)	0.8 (06-1.1)		32.8 (24.2-44.6)	0.7 (0.6-0.9)	
	Uninfected	49.1 (44.1-54.6)	Reference		44.7 (40.0-50.0)	Reference	
Other underlying illness[c]	Absent	46.9 (42.4-51.2)	Reference		42.3 (38.4-47.4)	Reference	
	Present	57.7 (32.8-101.6)	1.2 (0.7-2.1)		48.1 (25.9-89.4)	1.1 (0.7-1.8)	
Number of individuals in household	3-5	44.1 (38.3-50.8)	Reference		39.5 (34.0-45.8)	Reference	
	6-10	50.9 (43.9-59.0)	1.2 (0.9-1.5)		46.9 (40.2-54.7)	1.2 (0.9-1.5)	

	≥11	48.6 (33.6-70.4)	1.1 (0.6-1.9)	43.4 (29.3-64.3)	1.1 (0.7-1.8)
Crowding (people/sleeping room)	<2	42.1 (36.0-49.1)	Reference	38.1 (32.4-44.9)	Reference
	≥2	51.3 (45.2-58.3)	1.2 (0.9-1.5)	46.6 (40.1-53.3)	1.2 (0.9-1.5)

*Estimated using Poisson regression adjusted for clustering by site and household.

[a] Includes multiple episodes of RSV infection [b]Includes only the first episode of RSV infection [c]Self-reported history of asthma, lung disease, heart disease, stroke, spinal cord injury, epilepsy, organ transplant, immunosuppressive therapy, organ transplantation, cancer, liver disease, renal disease or diabetes

RR-Rate ratio

Additional variables evaluated but not found to be significant on univariate or multivariable analysis: Influenza vaccination, level of education, employment, use of alcohol, current or previous smoking, body mass index, current or previous tuberculosis, household income, mean indoor summer and winter respirable particulate matter, mean indoor summer and winter temperature

Supplementary table 6: Combinations of subgroup among individuals with single and repeat RSV infections within a season, at a rural and an urban site, South Africa, 2017-2018

**Combination of subgroups**	**1 episode n=325 (%)**	**2 episodes n=31 (%)**	**3 episodes n=3 (%)**
RSV A	119 (37)	4 (13)	0 (0)
RSV B	189 (58)	11 (35)	2 (67)
RSV A and B coinfection	3 (1)	0 (0)	0 (0)
RSV A/B	0 (0)	9 (29)	1 (33)
Subgroup not determined	14 (4)	0 (0)	0 (0)
RSV A/ Subgroup not determined	0 (0)	4 (13)	0 (0)
RSV B/ Subgroup not determined	0 (0)	2 (6)	0 (0)
RSV A and B coinfection/ Subgroup not determined	0 (0)	1 (3)	0 (0)

Supplementary table 7: Factors associated with being an index case of RSV within a household cluster at least once vs not being an index case among 1116 participants in the PHIRST study at a rural and an urban site, South Africa, 2017-2018 (analysis population all study participants) [a]

**Variable**		**Index case n/N (%)**	**Univariate RR (95% CI)**
Site	Rural	127/561 (23)	Reference
	Urban	116/555 (21)	0.9 (0.7-1.2)
Age group (years)	<1	10/22 (45)	6.1 (2.5-15.1)
	1-4	65/158 (41)	5.1 (3.2-8.2)
	5-12	69/302 (23)	2.2 (1.4-3.4)
	13-18	38/161 (24)	2.3 (1.4-3.8)
	19-44	35/291 (12)	Reference
	45-64	24/137 (18)	1.6 (0.9-2.7)
	≥65	2/45 (4)	0.3 (0.1-1.5)
Sex	Female	139/680 (20)	0.8 (0.6-1.1)
	Male	104/436 (24)	Reference
HIV	Uninfected	206/908 (23)	Reference
	Infected	29/167 (17)	0.7 (0.5-1.1)
Other underlying illness[b]	No	236/1089 (22)	Reference
	Yes	7/27 (26)	1.3 (0.5-3.0)

[a]Estimated using logistic regression adjusted for clustering by site and household. Of 1116 individuals in the cohort 243 were an index case for a cluster of RSV within a household at least once [b]Self-reported history of asthma, lung disease, heart disease, stroke, spinal cord injury, epilepsy, organ transplant, immunosuppressive therapy, organ transplantation, cancer, liver disease, renal disease or diabetes

Supplementary table 8: Characteristics associated with being an index case (vs non index case) for 400 infection episodes in household clusters of RSV infection in a rural and an urban site, South Africa, 2017-2018 (analysis population all individuals who had an RSV infection episode) [a]

**Variable**		**Index case n/N (%)**	**Univariate RR (95% CI)**
Site	Rural	138/209 (66)	Reference
	Urban	127/191 (66)	1.0 (0.6-1.6)
Age group (years)	<1	10/12 (83)	5.6 (1.0-31.7)
	1-4	71/95 (75)	3.1 (1.4-6.6)
	5-12	78/111 (70)	2.4 (1.2-4.8)
	13-18	41/65 (63)	1.7 (0.8-3.8)
	19-44	36/67 (54)	Reference
	45-64	26/44 (59)	1.3 (0.5-3.0)
	≥65	3/6 (50)	1.0 (0.7-2.2)
Sex	Female	150/240 (63)	0.6 (0.4-0.9)
	Male	115/160 (72)	Reference
HIV	Uninfected	224/339 (66)	Reference
	Infected	32/48 (67)	1.0 (0.5-1.9)
Other underlying illness[b]	No	257/388 (66)	Reference
	Yes	8/12 (67)	1.0 (0.3-3.7)

[a]Estimated using logistic regression adjusted for clustering by site and household, mixed infections counted as separate episodes [b]Self-reported history of asthma, lung disease, heart disease, stroke, spinal cord injury, epilepsy, organ transplant, immunosuppressive therapy, organ transplantation, cancer, liver disease, renal disease or diabetes

Supplementary table 9: Rates of RSV infections per 100 person-years by RSV subgroup by year and site, at a rural and an urban site, South Africa, 2017-2018[a]

Year	Site	RSV A		RSV B		RSV subgroup not determined	
		n	Rate[b] (95% CI)	n	Rate[b] (95% CI)	n	Rate[b] (95% CI)
2017-2018	Rural and urban	146	17.4 (14.8-20.46)	233	27.8 (24.4-31.6)	21	2.5 (1.6-3.8)
2017-2018	Rural	76	18.0 (14.4-22.6)	123	29.2 (24.5-34.8)	10	2.4 (1.3-4.4)
2017-2018	Urban	70	16.8 (13.3-21.2)	110	26.3 (21.9-31.8)	11	2.6 (1.5-4.8)
2017	Rural and urban	43	10.2 (7.6-13.8)	137	32.5 (27.5-38.5)	2	0.5 (0.1-1.9)
2017	Rural	40	18.6 (13.7-25.4)	47	21.9 (16.4-29.1)	0	0
2017	Urban	3	1.5 (0.5-4.5)	90	43.6 (35.5-53.6)	2	0.1 (0.2-3.9)
2018	Rural and urban	103	24.7 (20.3-29.9)	96	23.0 (18.8-28.1)	19	4.6 (2.9-7.1)
2018	Rural	36	17.4 (12.6-24.2)	76	36.8 (29.4-46.1)	10	4.8 (2.6-9.0)
2018	Urban	67	31.2 (25.0-40.3)	20	9.5 (6.1-14.7)	9	4.3 (2.2-8.2)

[a]Includes 4 episodes of mixed infection of RSV A and B [b]Incidence rate estimated as number of episodes divided by the person time under observation

Supplementary table 10: Rates[a] of RSV infections per 100 person-seasons by RSV subgroup and age group, at a rural and an urban site, South Africa, 2017-2018

**Age group (years)**	**RSV A**	**RSV B**	**RSV subgroup not determined**
<1	48.4 (24.2-96.7)	24.2 (9.1-64.4)	0
1-4	25.5 (17.9-36.5)	52.8 (41.1-67.7)	2.6 (0.8-7.9)
5-12	17.6 (12.9-23.9)	29.9 (23.5-37.9)	1.3 (0.4-4.1)
13-18	20.6 (13.9-30.4)	30.4 (22.1-42.0)	2.5 (0.8-7.7)
19-44	11.5 (7.8-17.0)	16.1 (11.6-22.4)	3.2 (1.5-6.8)
45-64	17.2 (10.9-27.4)	20.1 (13.1-30.9)	4.8 (2.0-11.5)
≥65	0	17.8 (8.0-39.6)	0

[a] Incidence rate estimated as number of episodes divided by the person time under observation

Supplementary table 11: Proportion of individuals seeking care and reporting absenteeism by number of symptoms reported, at a rural and an urban site, South Africa, 2017-2018

**Symptoms**	**1 symptom** n/N (%)	**≥2 symptoms (no ILI)** n/N (%)	**ILI** n/N (%)	**Any symptom** n/N (%)	**P[b]**
Seeking care	1/55 (2)	5/57 (9)	3/20 (15)	9/132 (7)	0.100
Absenteeism[a]	3/27 (11)	3/20 (15)	1/6 (17)	7/55 (13)	0.912

ILI – influenza-like illness [a]Absenteeism estimated among individuals attending school or work [b]Comparing 1 symptom, ≥2 symptoms (no ILI) and ILI using two sided Chi squared test

Supplementary table 12: Factors associated with generation interval among 94 individuals with interval <17 days at a rural and an urban site, South Africa, 2017-2018[a]

Variable		Interval (days) Mean (SD; range)	Univariate HR	Multivariable aHR
**Characteristics of the index case**				
Age group (years)	<1	5.4 (1.5; 4-7)	11.8 (3.0-47.3)	32.6 (7.7-137.1)
	1-4	7.7 (3.5; 2-14)	3.7 (1.1-11.2)	6.4 (2.3-18.0)
	5-12	9.0 (4.0; 1-16)	2.8 (0.9-8.1)	3.2 (1.2-8.5)
	13-18	6.7 (3.5; 2-13)	5.6 (1.6-20.4)	4.3 (1.3-13.8)
	19-44	8.3 (3.7; 3-13)	3.1 (1.0-10.3)	6.3 (1.9-20.5)
	45-64	14.0 (3.9; 7-16)	Reference	Reference
	≥65	Not estimated	Not estimated	Not estimated
Sex	Female	8.5 (4.3; 2-16)	0.9 (0.5-1.5)	
	Male	8.3 (3.7; 1-16)	Reference	
HIV	Infected	8.0 (4.2; 3-16)	1.1 (0.4-3.0)	
	Uninfected	8.3 (3.9; 1-16)	Reference	
	Unknown	16.0 (0; 16-16)	Not estimated	
Other underlying illness[b]	Absent	8.5 (4.0; 1-16)	Reference	
	Present	5.0 (1.4; 4-6)	4.6 (0.8-26.2)	
Number of symptoms	0	9.2 (4.2; 2-16)	Reference	
	1	7.0 (3.2; 3-12)	2.3 (0.8-6.4)	
	≥2	7.7 (3.8; 1-16)	1.5 (0.8-2.7)	
Duration of shedding (days)	<4	10.0 (5.4; 5-16)	Reference	
	4-10	7.9 (3.9; 2-16)	1.9 (0.5-6.5)	
	>10	8.3 (3.3; 1-16)	1.8 (0.5-6.5)	
Subgroup	A	7.4 (3.2; 2-15)	Reference	Reference
	B	8.8 (4.2; 1-16)	0.6 (0.4-1.2)	0.5 (0.3-0.8)
Minimum Ct value	<30	8.1 (3.8; 1-16)	1.4 (0.7-2.7)	
	30-37	9.3 (4.4; 2-16)	Reference	
**Characteristics of the household member**				
Age group (years)	<1	9.0 (0; 9-9)	1.1 (0.1-11.8)	0.9 (0.1-6.9)
	1-4	7.1 (4.8; 1-16)	1.9 (0.7-5.0)	2.0 (1.1-4.0)
	5-12	9.3 (3.8; 4-16)	Reference	Reference
	13-18	8.6 (4.4; 2-16)	1.1 (0.5-2.5)	0.8 (0.4-1.7)
	19-44	7.5 (3.3; 2-16)	1.9 (0.9-4.2)	1.7 (0.9-3.2)
	45-64	9.3 (3.6; 4-15)	1.1 (0.4-2.6)	0.6 (0.3-1.3)
	≥65	9.0 (6.1; 5-16)	1.2 (0.3-4.8)	1.2 (0.3-4.0)

Sex	Female	8.4 (3.9; 2-16)	1.0 (0.6-1.8)
	Male	8.4 (4.3; 1-16)	Reference
HIV	Infected	7.7 (3.7; 2-13)	1.2 (0.5-2.8)
	Uninfected	8.6 (4.1; 1-16)	Reference
	Unknown	6.0 (1.8; 4-8)	Not estimated

SD – Standard deviation [a]Estimated using Weibull accelerated failure time regression adjusted for clustering by site and household. Individuals with interval <17 days (n=96) included in the analysis. Samples were collected at 2 to 4 day intervals. Generation interval refers to the difference between the dates of the first positive PCR tests in the index case and in the secondary case each adjusted by adding a random number selected from a uniform distribution between 0 and 3 (inclusive). Overall mean interval 8.3 days (standard deviation 4.0; range 1-16 days). Hazard ration <1 corresponds to longer generation interval. [b]Self-reported history of asthma, lung disease, heart disease, stroke, spinal cord injury, epilepsy, organ transplant, immunosuppressive therapy, organ transplantation, cancer, liver disease, renal disease or diabetes

Additional factors evaluated but not found to be statistically significant include year, site, employment of index or contact, education level of index or contact, alcohol or smoking of index or contact, urine cotinine level of index or contact, underlying tuberculosis, other underlying illness of household contact; body mass index of index of index or household contact, receipt of influenza vaccine of index or contact, duration of shedding of index case, number of people in household, number of rooms, crowding, smoking inside the house, mean indoor summer and winter temperature, mean indoor summer and winter particulate matter.

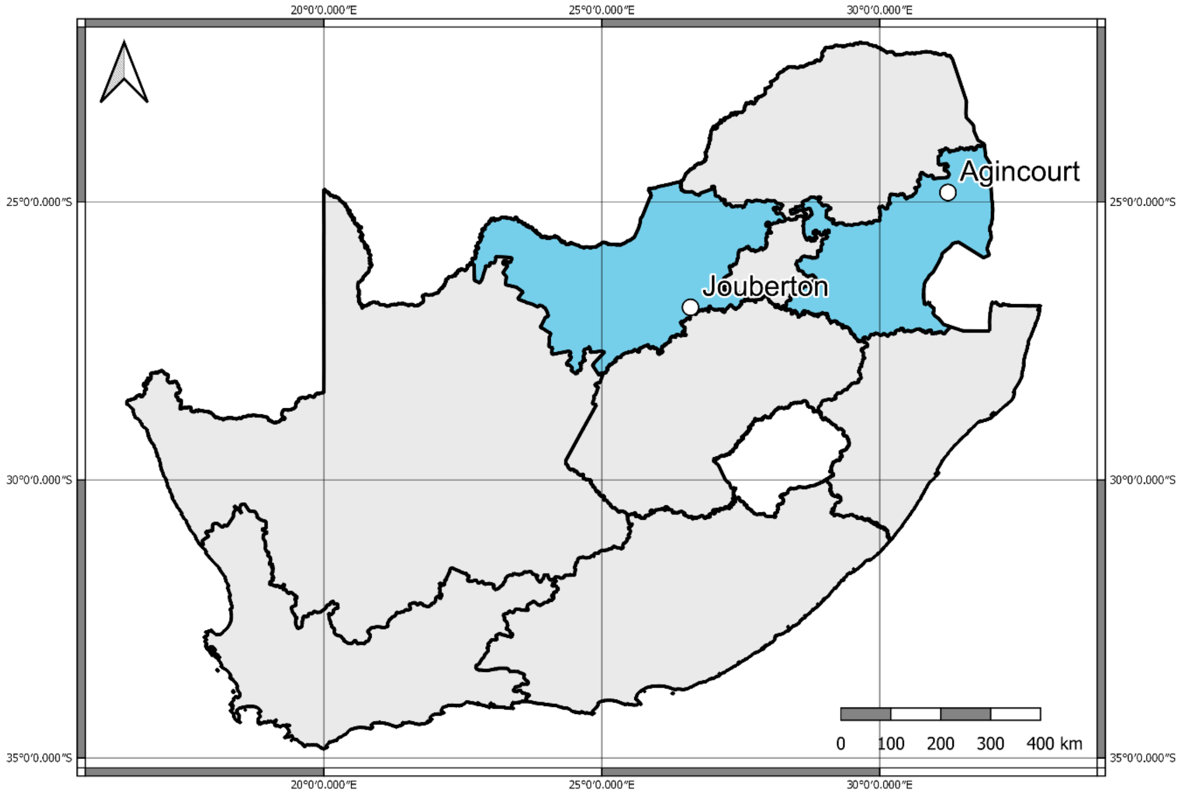

Supplementary figure 1: Location of rural (Agincourt, Mpumalanga Province) and urban (Jouberton, North West Province) study sites in South Africa. Generated using QGIS Geographic Information System (Open Source Geospatial Foundation Project).

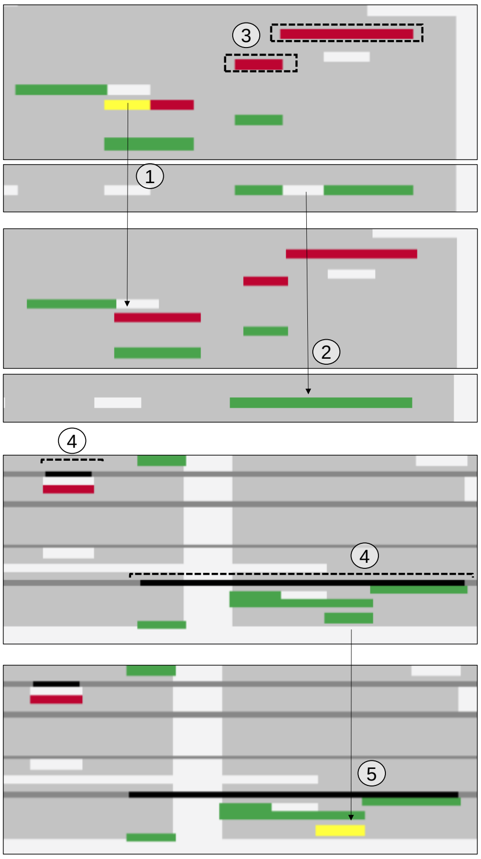

(1) For real-time reverse transcription polymerase chain reaction (rRT-PCR) positivity where subgroup was not determined we assigned a subgroup if the individual had a confirmed subgroup of the same RSV subgroup (A or B) within two preceding or following follow-up visits. Example, RSV subgroup not determined assigned to RSV A based on RSV A positivity at next visit

(2) We considered a new infection of the same subgroup in the same individual when the individual tested positive for the same subgroup >2 weeks of the last day of previous positivity; else we considered it as positivity from the same infection episode. Example, two positive RSV B swabs with a negative swab between updated to RSV B.

(3) We defined RSV infection episode as at least one nasopharyngeal swab rRT-PCR positive for RSV. Episode duration was estimated from the first to the last day of RSV rRT-PCR positivity with the same subgroup plus a random uniform distribution from 0-3 to account for the midpoint time from subsequent visits.

(4) A cluster was composed of all infections of the same subgroup within a household within an interval between infections of ≤2 mean serial intervals (3.5 days) including single infections within a household. Cluster duration was estimated as the interval from the first day of positivity of the first individual in a cluster to the last day of RSV positivity of the last individual plus a random uniform distribution from 0-3 on either end to account for the midpoint time from subsequent visits. Examples of clusters of different durations.

(5) For rRT-PCR positivity where subgroup was not determined we assigned a subgroup if the individual was part of a household cluster. Example, RSV subgroup not determined assigned to RSV B based on RSV B positivity within household for cluster

(6) The household cumulative infection risk (HCIR) was defined as cumulative number of all household members with RSV infection detected within a household cluster, divided by the total number of individuals participating in the study in the affected household, exclusive of the index case

(7) The primary/index case was defined as the first individual testing positive within a cluster.

(8) If two individuals tested positive in the same visit at the start of the cluster they were defined as co-primary cases and not included in the analysis of HCIR.

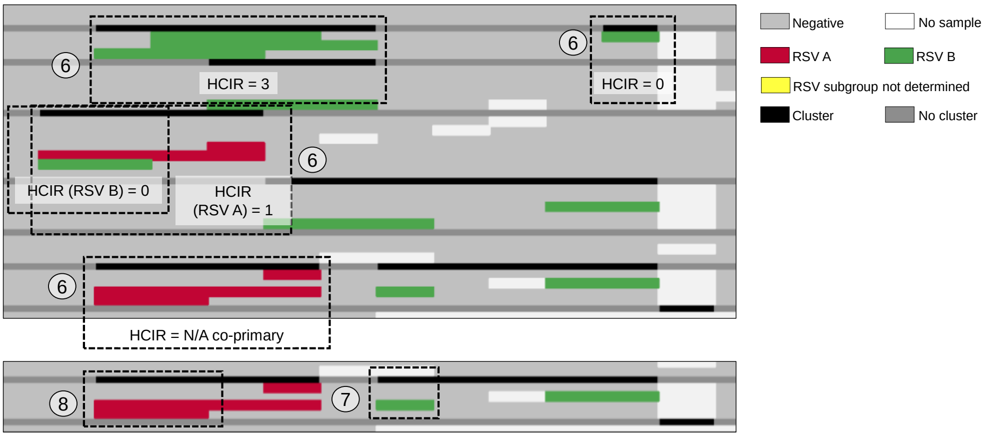

Supplementary figure 2 Description of the process for subgroup assignation and definition of episodes and clusters

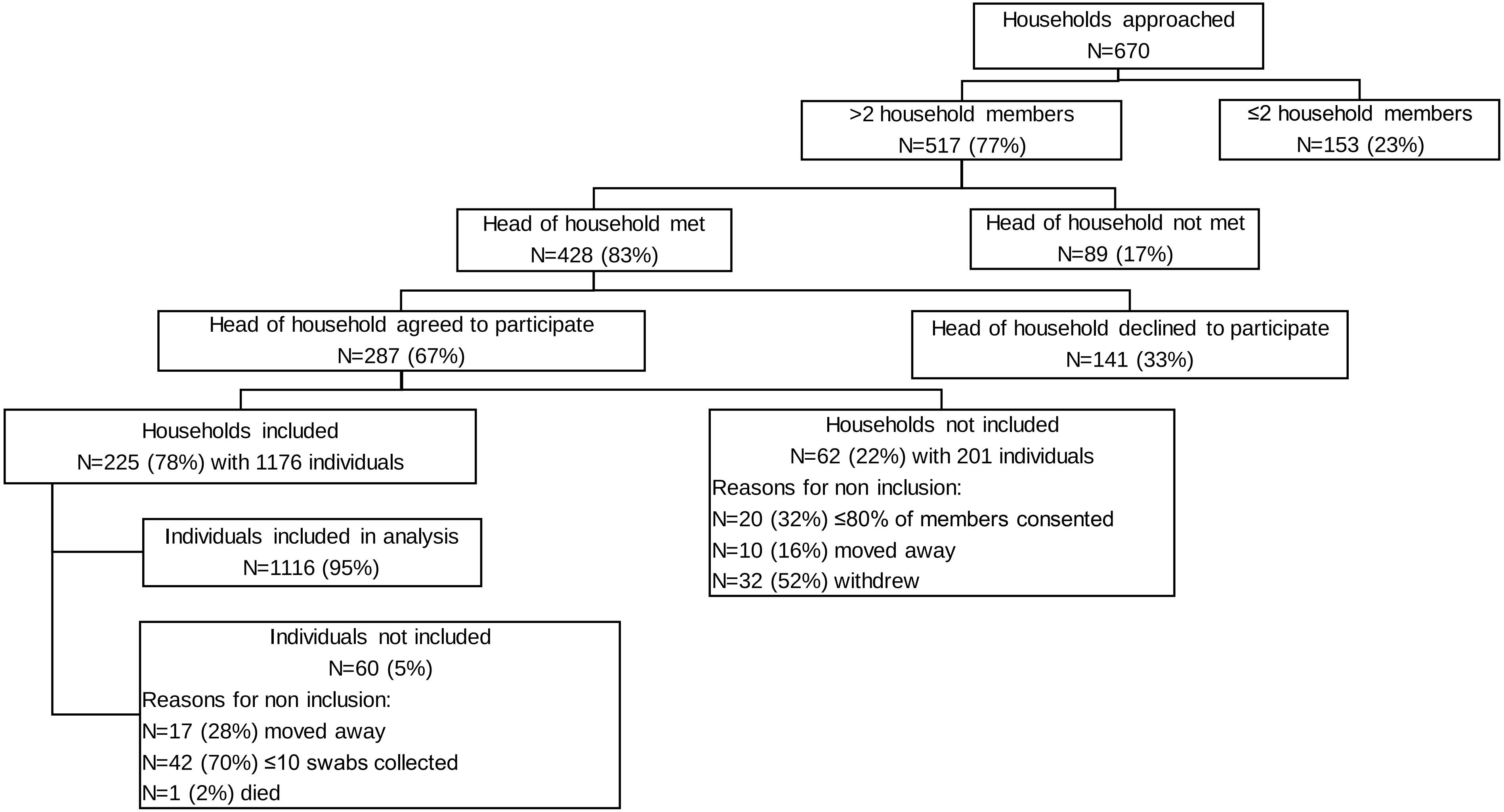

Supplementary figure 3: Flow chart of individuals included in the study, an urban and a rural site, South Africa, 2017-2018*

* Of 1116 individuals and 225 households included in the analysis, 108 (48%) households and 558 (50%) individuals were enrolled and followed up with twice-weekly swabbing in 2017 and 117 (52%) households and 558 (50%) individuals in 2018

2

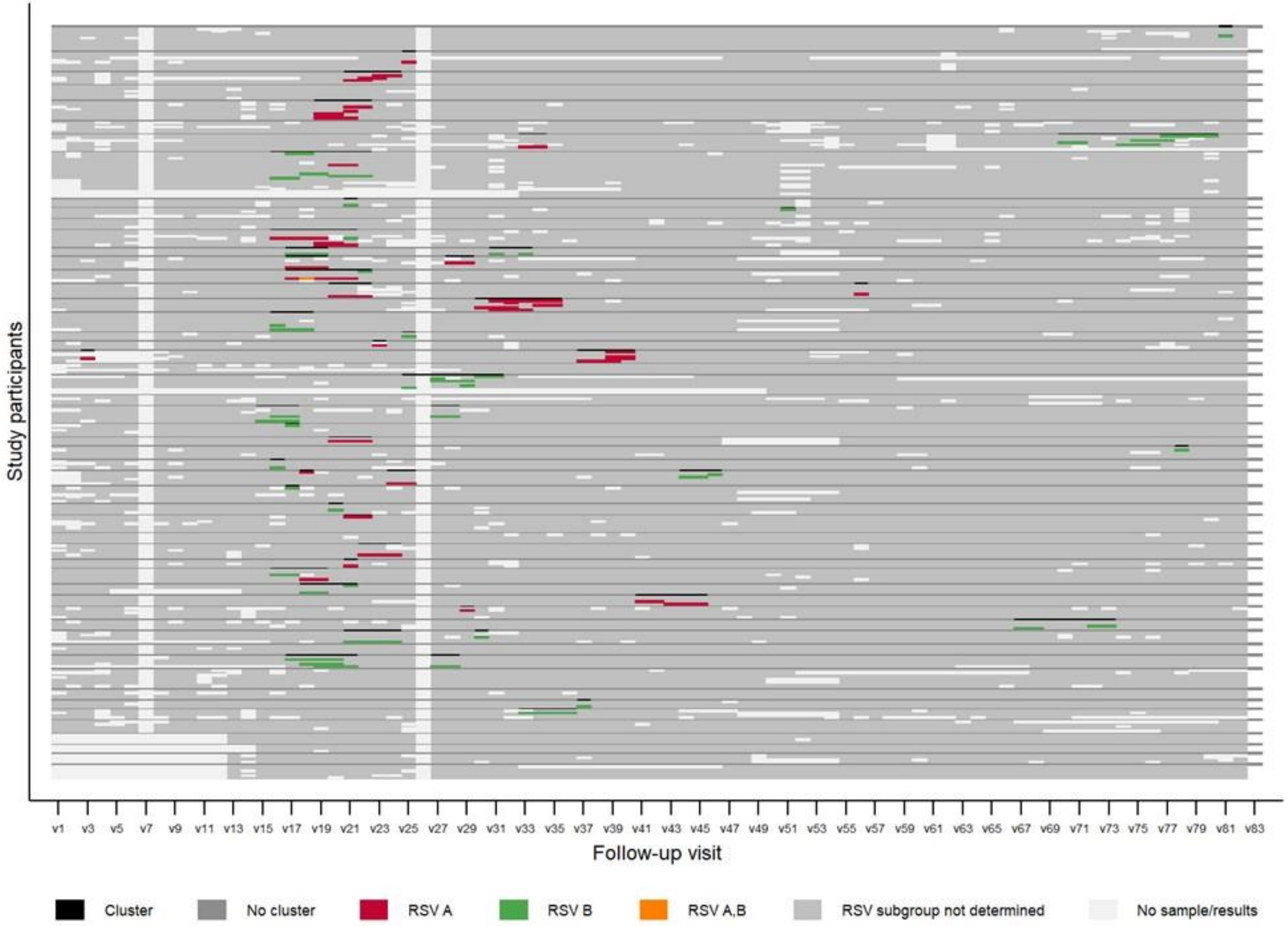

A rural 2017

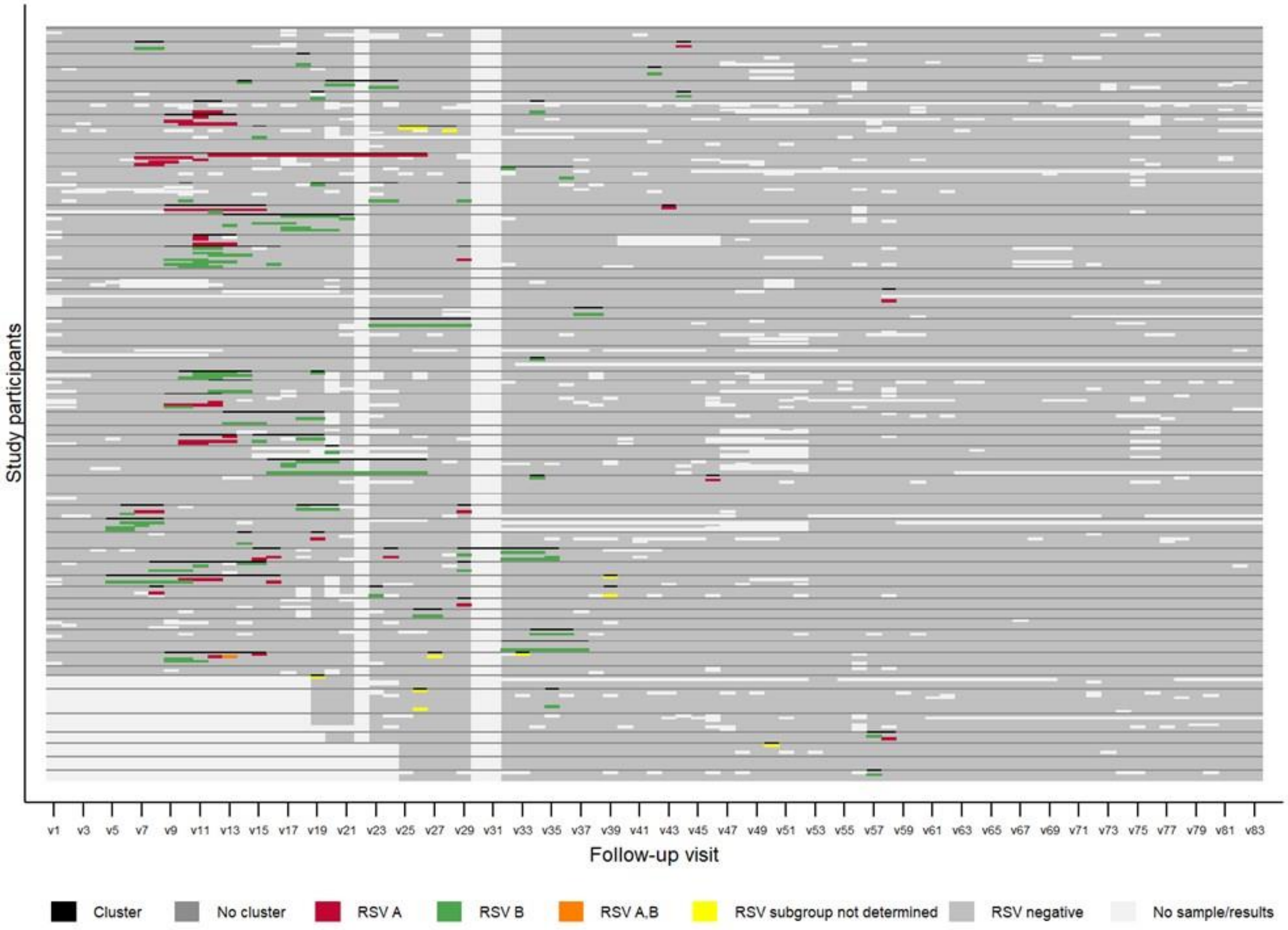

Study participants
v1 v3 v5 v7 v9 v11 v13 v15 v17 v19 v21 v23 v25 v27 v29 v31 v33 v35 v37 v39 v41 v43 v45 v47 v49 v51 v53 v55 v57 v59 v61 v63 v65 v67 v69 v71 v73 v75 v77 v79 v81 v83
Follow-up visit
Cluster
No cluster
RSV A
RSV B
RSV A,B
RSV subgroup not determined
RSV negative
No sample/results

B rural 2018

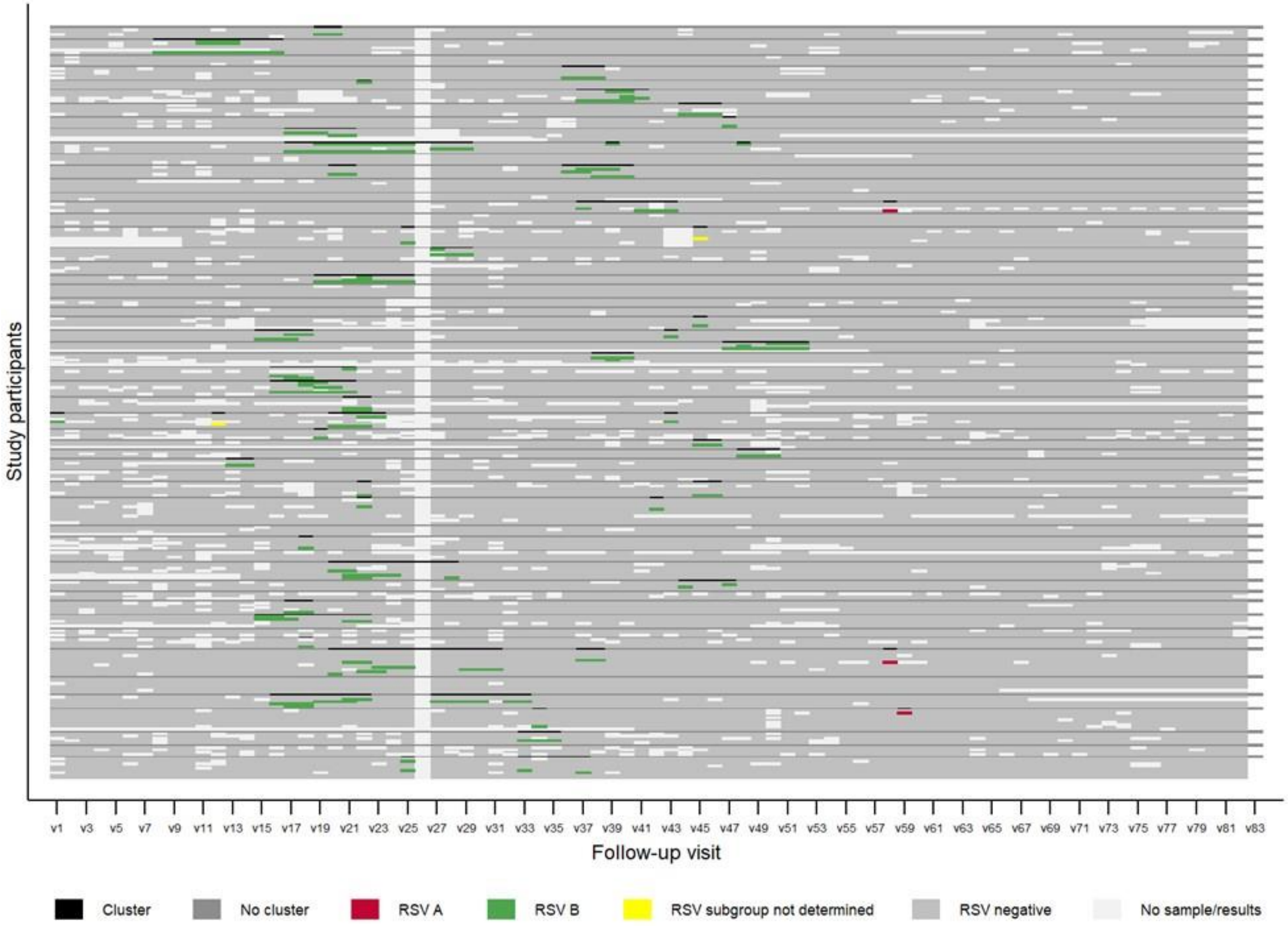

Study participants
v1
v3
v5
v7
v9
v11
v13
v15
v17
v19
v21
v23
v25
v27
v29
v31
v33
v35
v37
v39
v41
v43
v45
v47
v49
v51
v53
v55
v57
v59
v61
v63
v65
v67
v69
v71
v73
v75
v77
v79
v81
v83
Follow-up visit
Cluster
No cluster
RSV A
RSV B
RSV subgroup not determined
RSV negative
No sample/results

C Urban 2017

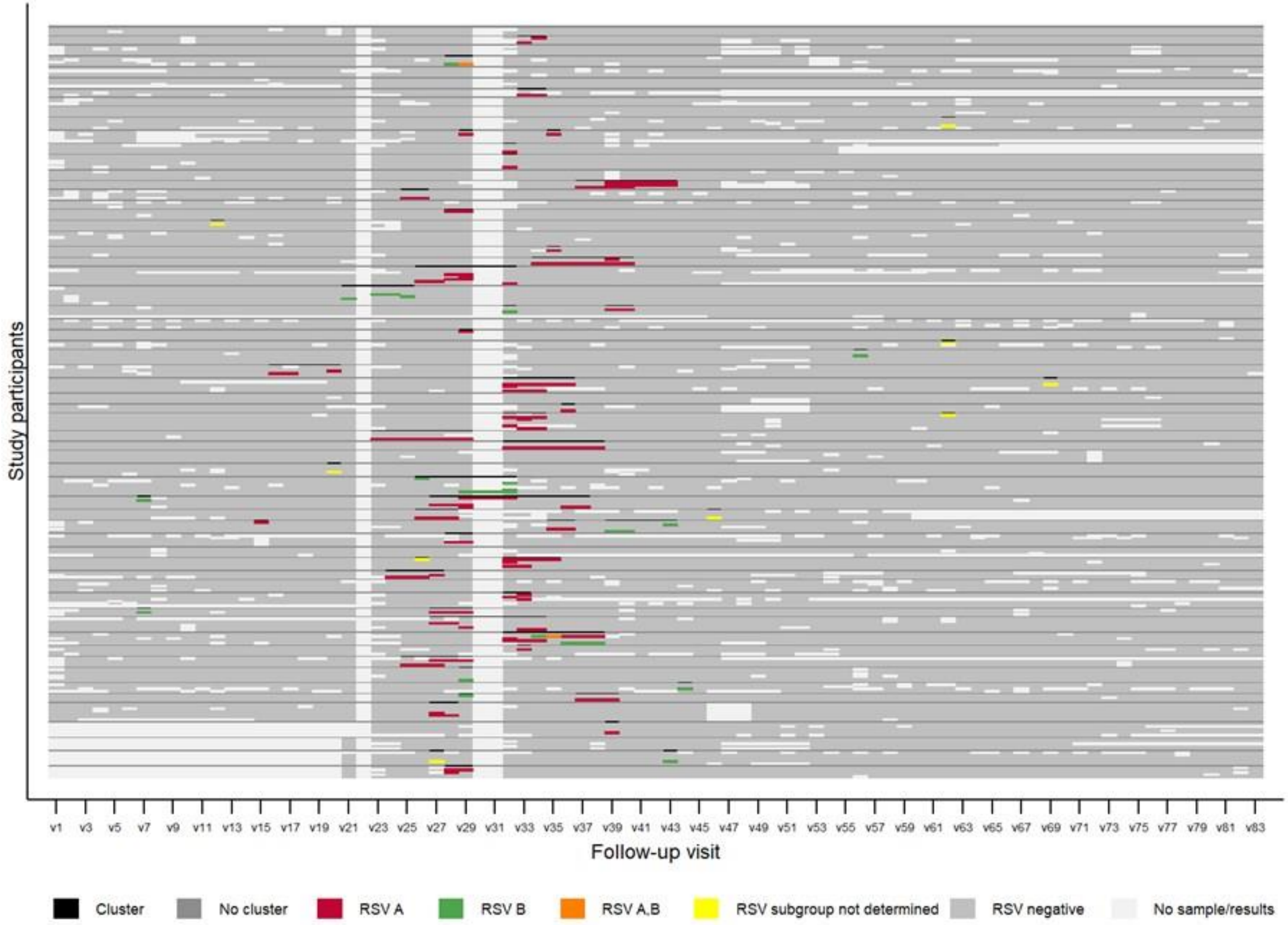
Study participants
v1 v3 v5 v7 v9 v11 v13 v15 v17 v19 v21 v23 v25 v27 v29 v31 v33 v35 v37 v39 v41 v43 v45 v47 v49 v51 v53 v55 v57 v59 v61 v63 v65 v67 v69 v71 v73 v75 v77 v79 v81 v83
Follow-up visit
Cluster
No cluster
RSV A
RSV B
RSV A,B
RSV subgroup not determined
RSV negative
No sample/results

D Urban 2018

Supplementary figure 4: RSV detection among study participants, and allocated clusters of infection. (a) rural site 2017 n=285 (b) rural site 2018 n=276 (c) urban site 2017 n=273 (b) urban site 2018 n=282. Columns are individual follow up visits and rows are individual participants. Each house is separated by a dark grey line on which clusters of infection are shaded black. The white, light grey and coloured horizontal lines each denote an individual with in a household. Each column indicates an individual follow up visit. Follow up visits are coloured white if no sample was tested, light grey if the sample tested negative for RSV and coloured according to the different RSV subgroups indicated in the legend if the nasopharyngeal swab tested positive for RSV. A high resolution version of this figure has been provided separately.

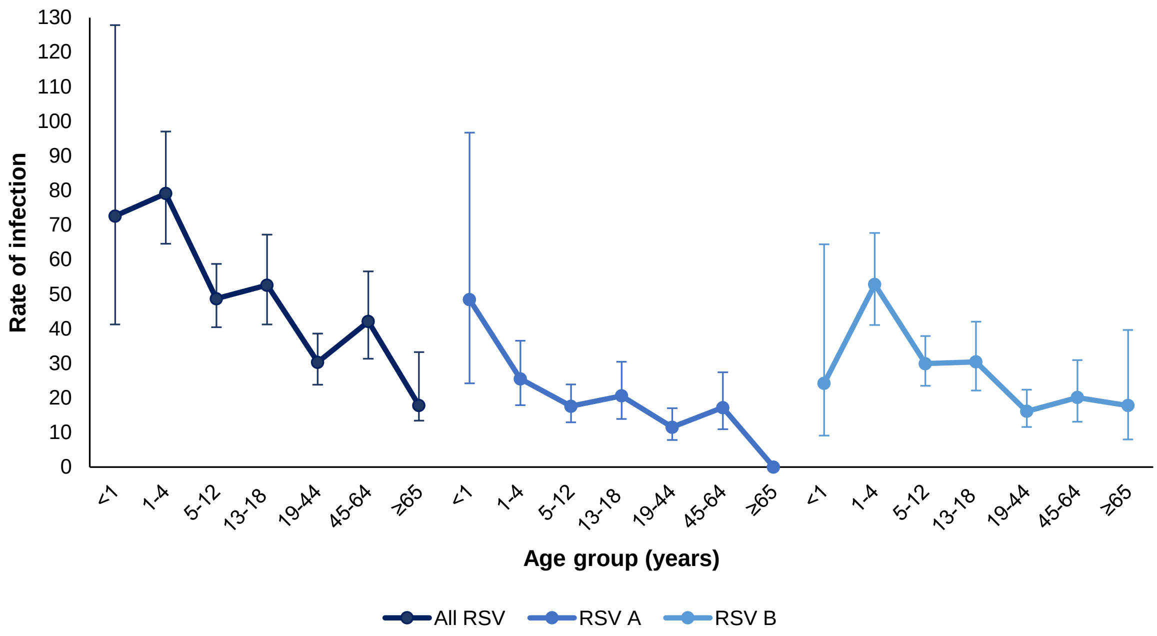

Supplementary figure 5: Rates of RSV infections per 100 person-years by age group and RSV subgroup, at a rural and an urban site, South Africa, 2017-2018

a

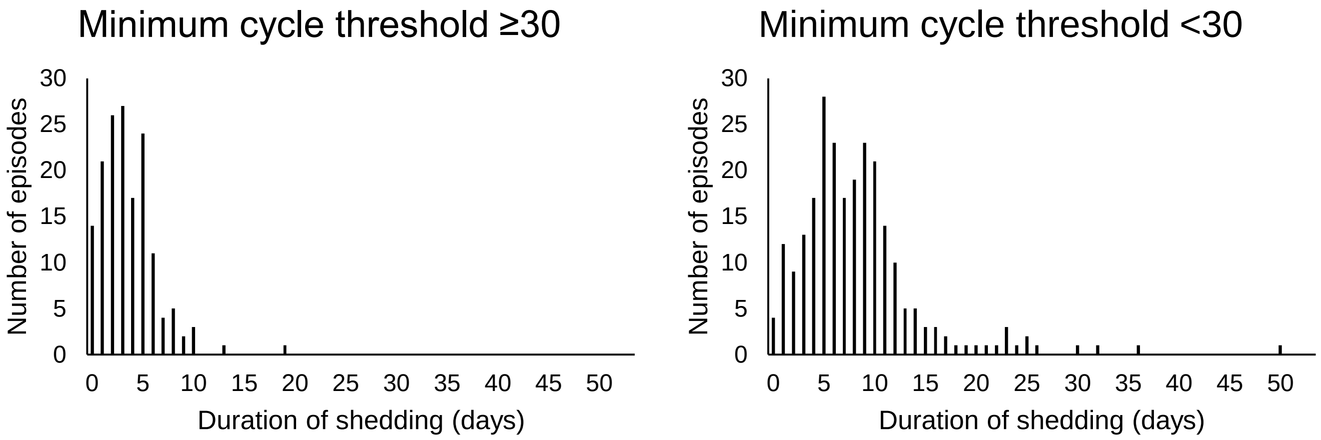

Minimum cycle threshold ≥30
Number of episodes
30
25
20
15
10
5
0
0 5 10 15 20 25 30 35 40 45 50
Duration of shedding (days)
Minimum cycle threshold <30
Number of episodes
30
25
20
15
10
5
0
0 5 10 15 20 25 30 35 40 45 50
Duration of shedding (days)

b

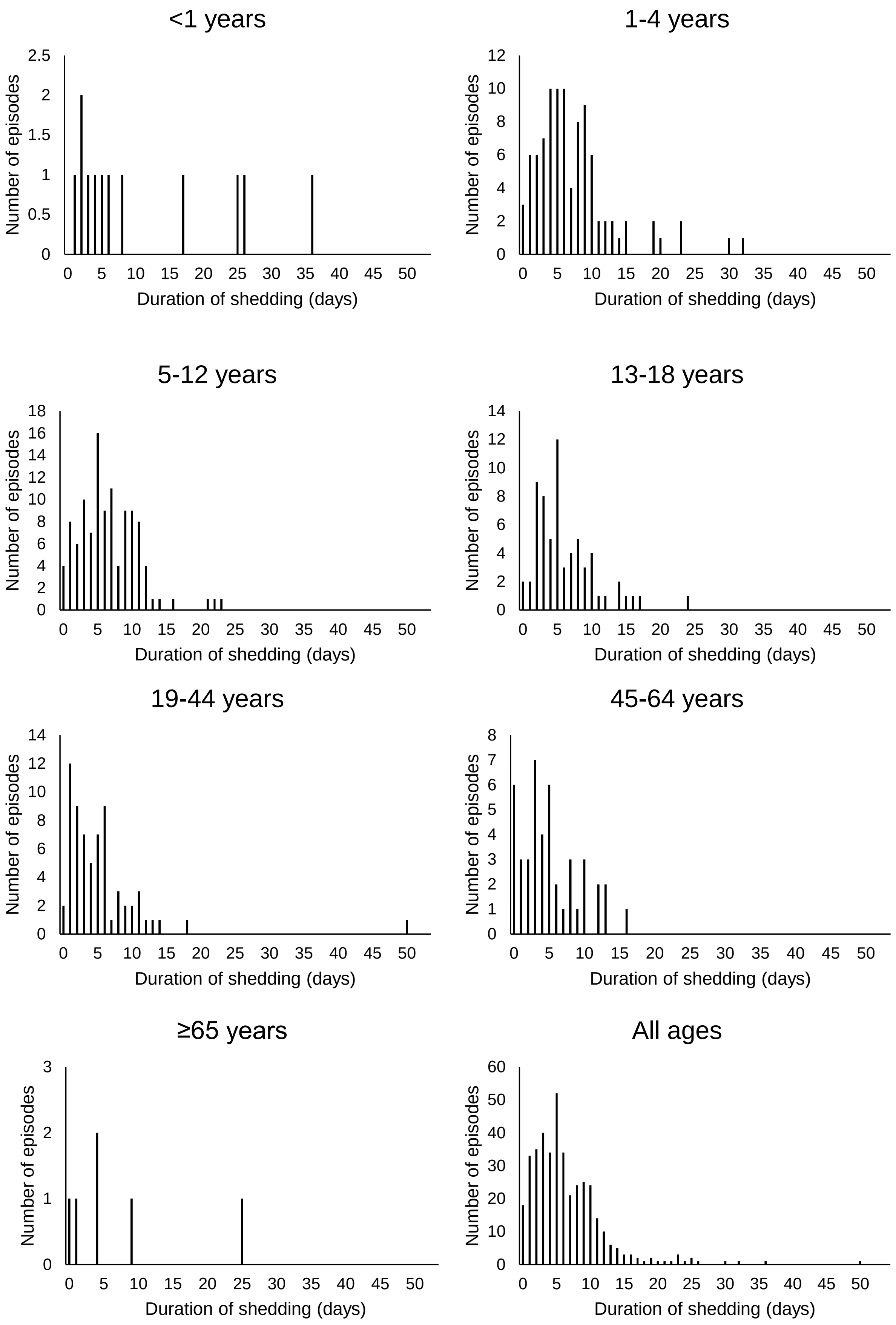

<1 years
1-4 years
5-12 years
13-18 years
19-44 years
45-64 years
≥65 years
All ages
Number of episodes
Duration of shedding (days)

c

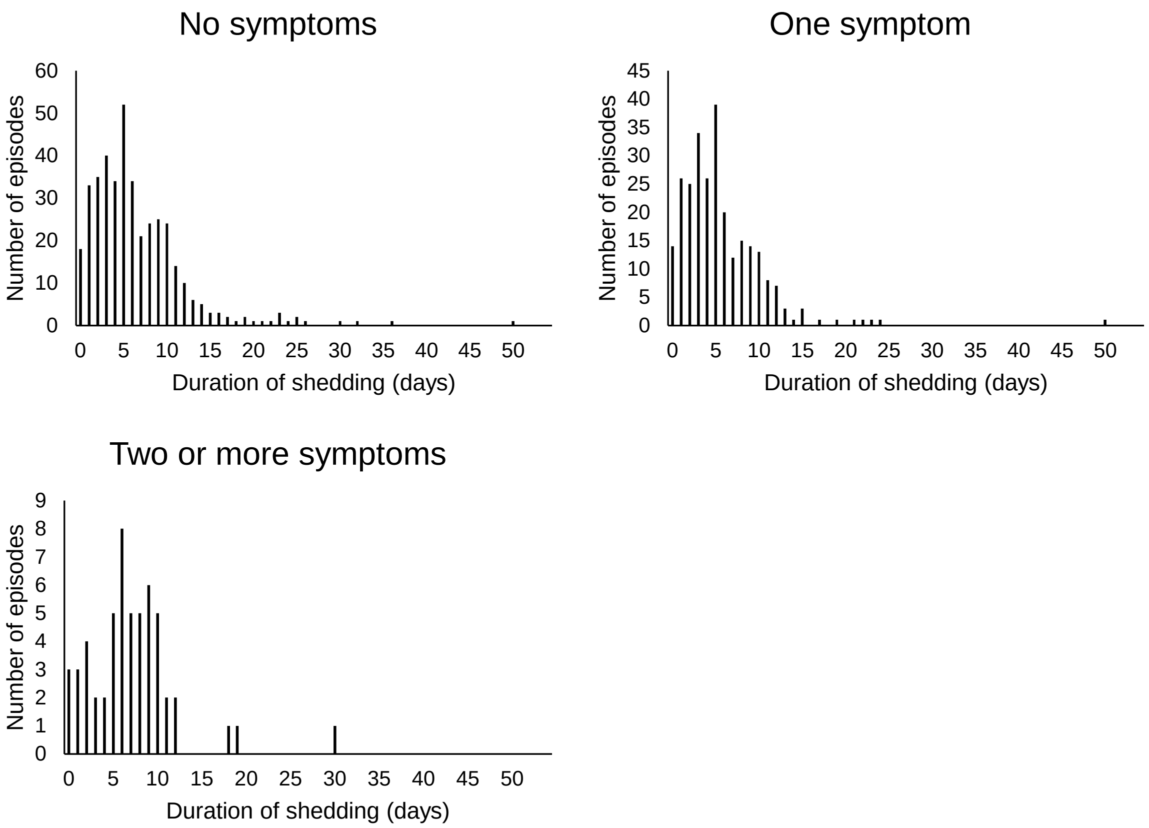

Supplementary figure 6: Duration of shedding of RSV detected on real-time polymerase chain reaction (rRT-PCR) at a rural and an urban site, South Africa, 2017-2018. Duration of shedding by (a) minimum cycle threshold (Ct) value (Ct <30 n=244, Ct≥30 n=156) (b) age group (<1 year n=12, 1-4 years n=95, 5-12 years n=111, 13-18 years n=65, 19-44 years n=67, 45-64 years n=44, ≥65 years n=6, all ages n=400) (c) number of symptoms (0 symptoms n=267, 1 symptom n=55, ≥2 symptoms n=78). Shedding duration of 0, indicates shedding for <1 day

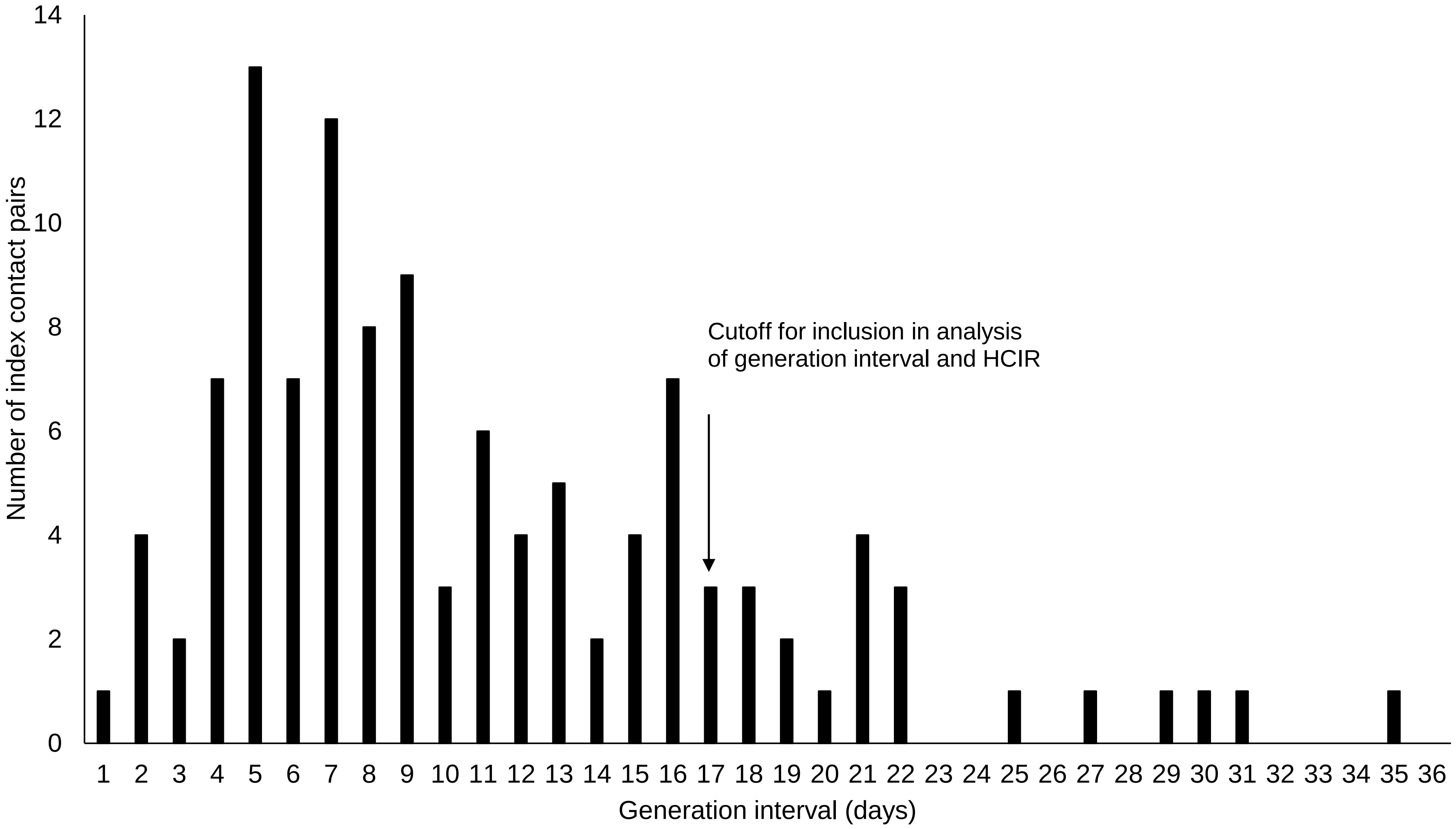

Supplementary figure 7: Interval between first RSV-positive real-time polymerase chain reaction (rRT-PCR) in the index case and first positive rRTPCR in household contacts (generation interval), at a rural and an urban site, South Africa, 2017-2018 (n=116)

STROBE Statement—Checklist of items that should be included in reports of ***cohort studies***

	Item No	Recommendation	Page No
**Title and abstract**	1	(*a*) Indicate the study's design with a commonly used term in the title or the abstract	1
		(*b*) Provide in the abstract an informative and balanced summary of what was done and what was found	3
**Introduction**			
Background/rationale	2	Explain the scientific background and rationale for the investigation being reported	4
Objectives	3	State specific objectives, including any prespecified hypotheses	4
**Methods**			
Study design	4	Present key elements of study design early in the paper	5-6
Setting	5	Describe the setting, locations, and relevant dates, including periods of recruitment, exposure, follow-up, and data collection	5-7 and supplement 26-28
Participants	6	(*a*) Give the eligibility criteria, and the sources and methods of selection of participants. Describe methods of follow-up	26 and supplement page 26-28
		(*b*) For matched studies, give matching criteria and number of exposed and unexposed	Not applicable
Variables	7	Clearly define all outcomes, exposures, predictors, potential confounders, and effect modifiers. Give diagnostic criteria, if applicable	5-7 and supplement page 26-28
Data sources/ measurement	8*	For each variable of interest, give sources of data and details of methods of assessment (measurement). Describe comparability of assessment methods if there is more than one group	5-7 and supplement page 26-28
Bias	9	Describe any efforts to address potential sources of bias	6-7 and 26-27
Study size	10	Explain how the study size was arrived at	5, 26
Quantitative variables	11	Explain how quantitative variables were handled in the analyses. If applicable, describe which groupings were chosen and why	5-7 and supplement page 28
Statistical methods	12	(*a*) Describe all statistical methods, including those used to control for confounding	5-7 and supplement page 28

		(*b*) Describe any methods used to examine subgroups and interactions<br>(*c*) Explain how missing data were addressed<br>(*d*) If applicable, explain how loss to follow-up was addressed<br>(*e*) Describe any sensitivity analyses	
**Results**			
Participants	13*	(a) Report numbers of individuals at each stage of study—eg numbers potentially eligible, examined for eligibility, confirmed eligible, included in the study, completing follow-up, and analysed<br>(b) Give reasons for non-participation at each stage<br>(c) Consider use of a flow diagram	8, 45
Descriptive data	14*	(a) Give characteristics of study participants (eg demographic, clinical, social) and information on exposures and potential confounders<br>(b) Indicate number of participants with missing data for each variable of interest<br>(c) Summarise follow-up time (eg, average and total amount)	8, 30-31
Outcome data	15*	Report numbers of outcome events or summary measures over time	8-9, 16-18, 33-42

Main results	16	(*a*) Give unadjusted estimates and, if applicable, confounder-adjusted estimates and their precision (eg, 95% confidence interval). Make clear which confounders were adjusted for and why they were included<br>(*b*) Report category boundaries when continuous variables were categorized<br>(*c*) If relevant, consider translating estimates of relative risk into absolute risk for a meaningful time period	8-9, 16-18, 33-42
Other analyses	17	Report other analyses done—eg analyses of subgroups and interactions, and sensitivity analyses	8, 39-40
**Discussion**			
Key results	18	Summarise key results with reference to study objectives	10
Limitations	19	Discuss limitations of the study, taking into account sources of potential bias or imprecision. Discuss both direction and magnitude of any potential bias	10-11
Interpretation	20	Give a cautious overall interpretation of results considering objectives, limitations, multiplicity of analyses, results from similar studies, and other relevant evidence	10-12
Generalisability	21	Discuss the generalisability (external validity) of the study results	10-11
**Other information**			
Funding	22	Give the source of funding and the role of the funders for the present study and, if applicable, for the original study on which the present article is based	13

*Give information separately for exposed and unexposed groups.

Note: An Explanation and Elaboration article discusses each checklist item and gives methodological background and published examples of transparent reporting. The STROBE checklist is best used in conjunction with this article (freely available on the Web sites of PLoS Medicine at http://www.plosmedicine.org/, Annals of Internal Medicine at http://www.annals.org/, and Epidemiology at http://www.epidem.com/). Information on the STROBE Initiative is available at http://www.strobe-statement.org.

1

The PHIRST group

Name	Affiliation
Amelia Buys	Centre for Respiratory Diseases and Meningitis, National Institute for Communicable Diseases of the National Health Laboratory Service, Johannesburg, South Africa.
Angie Mathee	Environment and Health Research Unit, South African Medical Research Council, Johannesburg, South Africa.
Anne von Gottberg	Centre for Respiratory Diseases and Meningitis, National Institute for Communicable Diseases of the National Health Laboratory Service, Johannesburg, South Africa. School of Pathology, Faculty of Health Sciences, University of the Witwatersrand
Brigitte Language	Unit for Environmental Science and Management, School of Geo- and Spatial Science, North-West University, Potchefstroom, South Africa.
Cheryl Cohen	Centre for Respiratory Diseases and Meningitis, National Institute for Communicable Diseases of the National Health Laboratory Service, Johannesburg, South Africa. School of Public Health, Faculty of Health Sciences, University of the Witwatersrand, Johannesburg, South Africa
Lorens Maake	Centre for Respiratory Diseases and Meningitis, National Institute for Communicable Diseases of the National Health Laboratory Service, Johannesburg, South Africa. School of Public Health, Faculty of Health Sciences, University of the Witwatersrand, Johannesburg
Floidy Wafawanaka	MRC/Wits Rural Public Health and Health Transitions Research Unit (Agincourt), Faculty of Health Sciences, School of Public Health, University of the Witwatersrand, Johannesburg, South Africa.
Florette Treurnicht	Centre for Respiratory Diseases and Meningitis, National Institute for Communicable Diseases of the National Health Laboratory Service, Johannesburg, South Africa. School of Pathology, Faculty of Health Sciences, University of the Witwatersrand
Jackie Kleynhans	Centre for Respiratory Diseases and Meningitis, National Institute for Communicable Diseases of the National Health Laboratory Service, Johannesburg, South Africa. School of Public Health, Faculty of Health Sciences, University of the Witwatersrand, Johannesburg, South Africa
Jocelyn Moyes	Centre for Respiratory Diseases and Meningitis, National Institute for Communicable Diseases of the National Health Laboratory Service, Johannesburg, South Africa. School of Public Health, Faculty of Health Sciences, University of the Witwatersrand, Johannesburg, South Africa
Kathleen Kahn	MRC/Wits Rural Public Health and Health Transitions Research Unit (Agincourt), School of Public Health, Faculty of Health Sciences, University of the Witwatersrand, Johannesburg, South Africa.
Katlego Mothlaoleng	Perinatal HIV Research Unit, MRC Soweto Matlosana Collaborating Centre for HIV/AIDS and TB, University of the Witwatersrand, Johannesburg, South Africa.
Limakatso Lebina	Perinatal HIV Research Unit, MRC Soweto Matlosana Collaborating Centre for HIV/AIDS and TB, University of the Witwatersrand, Johannesburg, South Africa.
Maimuna Carrim	Centre for Respiratory Diseases and Meningitis, National Institute for Communicable Diseases of the National Health Laboratory Service, Johannesburg, South Africa.
Meredith L McMorrow	Formerly Influenza Division, Centers for Disease Control and Prevention (CDC), Atlanta, Georgia, United States of America (USA); Influenza Program, Centers for Disease Control and Prevention, Pretoria, South Africa; currently, Coronavirus and Other Respiratory Viruses Division (proposed), CDC, Atlanta, GA USA.
Neil A Martinson	Perinatal HIV Research Unit, MRC Soweto Matlosana Collaborating Centre for HIV/AIDS and TB, University of the Witwatersrand, Johannesburg, South Africa. DST/NRF Centre of Excellence for Biomedical Tuberculosis

	Research, University of the Witwatersrand, Johannesburg, South Africa. Johns Hopkins University Center for TB Research, Baltimore, Maryland, United States of America.
Nicole Wolter	Centre for Respiratory Diseases and Meningitis, National Institute for Communicable Diseases of the National Health Laboratory Service, Johannesburg, South Africa. School of Pathology, Faculty of Health Sciences, University of the Witwatersrand
Orienka Hellferscee	Centre for Respiratory Diseases and Meningitis, National Institute for Communicable Diseases of the National Health Laboratory Service, Johannesburg, South Africa. School of Pathology, Faculty of Health Sciences, University of the Witwatersrand
Ryan G Wagner	MRC/Wits Rural Public Health and Health Transitions Research Unit (Agincourt), School of Public Health, Faculty of Health Sciences, University of the Witwatersrand, Johannesburg, South Africa.
Stefano Tempia	Influenza Division, Centers for Disease Control and Prevention, Atlanta, Georgia, United States of America. Influenza Program, Centers for Disease Control and Prevention, Pretoria, South Africa. School of Public Health, Faculty of Health Sciences, University of the Witwatersrand, Johannesburg, South Africa. MassGenics, Atlanta, Georgia, United States of America.
Stuart Piketh	Unit for Environmental Science and Management, School of Geo- and Spatial Science, North-West University, Potchefstroom, South Africa.
Thulisa Mkhencele	Centre for Respiratory Diseases and Meningitis, National Institute for Communicable Diseases of the National Health Laboratory Service, Johannesburg, South Africa.
F. Xavier Gómes-Olivé	MRC/Wits Rural Public Health and Health Transitions Research Unit (Agincourt), School of Public Health, Faculty of Health Sciences, University of the Witwatersrand, Johannesburg, South Africa.

References

1. Li Y, Wang X, Blau DM, et al. Global, regional, and national disease burden estimates of acute lower respiratory infections due to respiratory syncytial virus in children younger than 5 years in 2019: a systematic analysis. *Lancet*. 2022;399(10340):2047-2064. doi:10.1016/s0140-6736(22)00478-0

2. Shi T, Denouel A, Tietjen AK, et al. Global disease burden estimates of respiratory syncytial virus-associated acute respiratory infection in older adults in 2015: A systematic review and meta-analysis. *J Infect Dis*. 2021;222(Suppl 7):S577-S583. doi:10.1093/INFDIS/JIZ059

3. Mazur N, Terstappen J, Baral R, et al. Respiratory syncytial virus prevention within reach: the vaccine and monoclonal antibody landscape. *Lancet Infect Dis*. Published online August 8, 2022. doi:10.1016/S1473-3099(22)00291-2

4. Munywoki PK, Koech DC, Agoti CN, et al. The source of respiratory syncytial virus infection in infants: a household cohort study in rural Kenya. *J Infect Dis*. 2014;209(11):1685-1692. doi:10.1093/infdis/jit828

5. Kahn K, Collinson MA, Xavier Gómez-olivé F, et al. Profile: Agincourt health and socio-demographic surveillance system. *Int J Epidemiol*. 2012;41(4):988-1001. doi:10.1093/ije/dys115

6. Garenne M, Collinson MA, Kabudula CW, Gómez-Olivé FX, Kahn K, Tollman S. Completeness of birth and death registration in a rural area of South Africa: the Agincourt health and demographic surveillance, 1992-2014. *Glob Health Action*. 2016;9(1):32795. doi:10.3402/gha.v9.32795

7. Wong KKL, von Mollendorf C, Martinson N, et al. Healthcare utilization for common infectious disease syndromes in Soweto and Klerksdorp, South Africa. *Pan Afr Med J*. 2018;30:271. doi:10.11604/pamj.2018.30.271.14477

8. Cohen C, McMorrow ML, Martinson NA, et al. Cohort profile: A Prospective Household cohort study of Influenza, Respiratory syncytial virus and other respiratory pathogens community burden and Transmission dynamics in South Africa, 2016-2018. *Influenza Other Respi Viruses*. 2021;15(6):789-803. doi:10.1111/irv.12881

9. Cohen C, Kleynhans J, Moyes J, et al. Asymptomatic transmission and high community burden of seasonal influenza in an urban and a rural community in South Africa, 2017-18 (PHIRST): a population cohort study. *Lancet Glob Heal*. 2021;9(6):e863-e874. doi:10.1016/S2214-109X(21)00141-8

10. van de Pol AC, Wolfs TFW, van Loon AM, et al. Molecular quantification of

respiratory syncytial virus in respiratory samples: reliable detection during the initial phase of infection. *J Clin Microbiol*. 2010;48(10):3569-3574. doi:10.1128/JCM.00097-10

11. Hu A, Colella M, Tam JS, Rappaport R, Cheng SM. Simultaneous detection, subgrouping, and quantitation of respiratory syncytial virus A and B by real-time PCR. *J Clin Microbiol*. 2003;41(1):149-154. doi:10.1128/jcm.41.1.149-154.2003

12. Munywoki PK, Koech DC, Agoti CN, et al. Frequent Asymptomatic Respiratory Syncytial Virus Infections During an Epidemic in a Rural Kenyan Household Cohort. *J Infect Dis*. 2015;212(11):1711-1718. doi:10.1093/infdis/jiv263

13. Hall CB, Geiman JM, Biggar R, Kotok DI, Hogan PM, Douglas RG. Respiratory Syncytial Virus Infections within Families. *N Engl J Med*. 1976;294(8):414-419. doi:10.1056/nejm197602192940803

14. Valley-Omar Z, Tempia S, Hellferscee O, et al. Human respiratory syncytial virus diversity and epidemiology among patients hospitalized with severe respiratory illness in South Africa, 2012-2015. *Influenza Other Respi Viruses*. 2022;16(2):222-235. doi:10.1111/irv.12905

15. Hornsleth A, Klug B, Nir M, et al. Severity of respiratory syncytial virus disease related to type and genotype of virus and to cytokine values in nasopharyngeal secretions. *Pediatr Infect Dis J*. 1998;17(12):1114-1121. doi:10.1097/00006454-199812000-00003

16. Laham FR, Mansbach JM, Piedra PA, et al. Clinical Profiles of Respiratory Syncytial Virus Subtypes A AND B Among Children Hospitalized with Bronchiolitis. *Pediatr Infect Dis J*. 2017;36(8):808-810. doi:10.1097/INF.0000000000001596

17. Martinello RA, Chen MD, Weibel C, Kahn JS. Correlation between respiratory syncytial virus genotype and severity of illness. *J Infect Dis*. 2002;186(6):839-842. doi:10.1086/342414

18. Moyes J, Walaza S, Pretorius M, et al. Respiratory syncytial virus in adults with severe acute respiratory illness in a high HIV prevalence setting. *J Infect*. 2017;75(4). doi:10.1016/j.jinf.2017.06.007

19. McMorrow ML, Tempia S, Walaza S, et al. The Role of Human Immunodeficiency Virus in Influenza- and Respiratory Syncytial Virus-associated Hospitalizations in South African Children, 2011-2016. *Clin Infect Dis*. 2019;68(5):773-780. doi:10.1093/cid/ciy532

20. Shi T, Balsells E, Singleton R, et al. Risk factors for respiratory syncytial virus associated with acute lower respiratory infection in children under five years : Systematic review and meta – analysis. 2015;5(2). doi:10.7189/jogh.05.020416

21. Munywoki PK, Koech DC, Agoti CN, et al. Influence of age, severity of infection, and

co-infection on the duration of respiratory syncytial virus (RSV) shedding. *Epidemiol Infect*. 2015;143(4):804-812. doi:10.1017/S0950268814001393

22. Falsey AR, Walsh EE. Respiratory Syncytial Virus Infection in Adults. *Clin Microbiol Rev*. 2000;13(3):371-384. doi:10.1128/CMR.13.3.371

23. Rha B, Dahl RM, Moyes J, et al. Performance of Surveillance Case Definitions in Detecting Respiratory Syncytial Virus Infection among Young Children Hospitalized with Severe Respiratory Illness - South Africa, 2009-2014. *J Pediatric Infect Dis Soc*. 2019;8(4):325-333. doi:10.1093/jpids/piy055

24. Moyes J, Tempia S, Walaza S, et al. The burden of RSV-associated illness in children aged <5 years, South Africa, 2011 to 2016. *medRxiv Prepr Serv Heal Sci*. Published online 2022. https://www.medrxiv.org/content/10.1101/2022.06.20.22276680v1

25. Moyes J, Tempia S, Walaza S, et al. The economic burden of RSV-associated illness in children aged <5 years, South Africa 2011-2016. *medRxiv Prepr Serv Heal Sci*. Published online 2022. https://www.medrxiv.org/content/10.1101/2022.06.20.22276632v1

26. Hall CB, Douglas RG, Geiman JM. Respiratory syncytial virus infections in infants: Quantitation and duration of shedding. *J Pediatr*. 1976;89(1):11-15. doi:10.1016/S0022-3476(76)80918-3

27. Hall CB, Long CE, Schnabel KC. Respiratory syncytial virus infections in previously healthy working adults. *Clin Infect Dis*. 2001;33(6):792-796. doi:10.1086/322657

28. Okiro EA, White LJ, Ngama M, Cane PA, Medley GF, Nokes DJ. Duration of shedding of respiratory syncytial virus in a community study of Kenyan children. *BMC Infect Dis*. 2010;10. doi:10.1186/1471-2334-10-15

29. Cohen C, Kleynhans J, von Gottberg A, et al. SARS-CoV-2 incidence, transmission, and reinfection in a rural and an urban setting: results of the PHIRST-C cohort study, South Africa, 2020-21. *Lancet Infect Dis*. 2022;22(6):821-834. doi:10.1016/S1473-3099(22)00069-X

30. Crowcroft NS, Zambon M, Harrison TG, Mok Q, Heath P, Miller E. Respiratory syncytial virus infection in infants admitted to paediatric intensive care units in London, and in their families. *Eur J Pediatr*. 2008;167(4):395-399. doi:10.1007/s00431-007-0509-9

31. Berglund B. Respiratory Syncytial Virus Infections in Families: A Study of Family Members of Children Hospitalized for Acute Respiratory Disease. *Acta Pædiatrica*. 1967;56(4):395-404. doi:10.1111/j.1651-2227.1967.tb15398.x

32. Badger GF, Dingle JH, Feller AE, Hodges RG, Jordan WS, Rammelkamp CH. A study of illness in a group of cleveland families: III. Introduction of respiratory infections into families. *Am J Epidemiol*. 1953;58(1):41-46. doi:10.1093/oxfordjournals.aje.a119589

33. Stensballe L, Poulsen A, Nante E, et al. Mothers may transmit RSV infection more easily or severely to sons than daughters: Community study from Guinea-Bissau. *Scand J Infect Dis*. 2004;36(4):291-295. doi:10.1080/00365540410019589

34. Wong KKL, von Mollendorf C, Martinson N, et al. Healthcare utilization for common infectious disease syndromes in Soweto and Klerksdorp, South Africa. *Pan Afr Med J*. 2018;30:271. doi:10.11604/pamj.2018.30.271.14477

35. Cohen C, McMorrow ML, Martinson NA, et al. Cohort profile: A Prospective Household cohort study of Influenza, Respiratory syncytial virus and other respiratory pathogens community burden and Transmission dynamics in South Africa, 2016-2018. *Influenza Other Respi Viruses*. 2021;15(6):789-803. doi:10.1111/irv.12881

36. Fast-track diagnostics. *Reference Validation FTD FLU/HRSV*.; 2014. http://www.fast-trackdiagnostics.com/human-line/products/ftd-fluhrsv/
